# Calcium signaling mediates proliferation of the precursor cells that give rise to the ciliated left-right organizer in the zebrafish embryo

**DOI:** 10.3389/fmolb.2023.1292076

**Published:** 2023-12-12

**Authors:** Osama Abdel-Razek, Amanda Marzouk, Madison MacKinnon, Edward T. Guy, Sonny A. Pohar, Emily Zhushma, Junjie Liu, Isabel Sia, Jason J. Gokey, Hwee Goon Tay, Jeffrey D. Amack

**Affiliations:** ^1^ Department of Cell and Developmental Biology, State University of New York Upstate Medical University, Syracuse, NY, United States; ^2^ BioInspired Syracuse: Institute for Material and Living Systems, Syracuse, NY, United States

**Keywords:** embryonic development, organ laterality, birth defects, left-right organizer, cilia, Ca^2+^ signaling, cell cycle, zebrafish

## Abstract

Several of our internal organs, including heart, lungs, stomach, and spleen, develop asymmetrically along the left-right (LR) body axis. Errors in establishing LR asymmetry, or laterality, of internal organs during early embryonic development can result in birth defects. In several vertebrates—including humans, mice, frogs, and fish—cilia play a central role in establishing organ laterality. Motile cilia in a transient embryonic structure called the “left-right organizer” (LRO) generate a directional fluid flow that has been proposed to be detected by mechanosensory cilia to trigger asymmetric signaling pathways that orient the LR axis. However, the mechanisms that control the form and function of the ciliated LRO remain poorly understood. In the zebrafish embryo, precursor cells called dorsal forerunner cells (DFCs) develop into a transient ciliated structure called Kupffer’s vesicle (KV) that functions as the LRO. DFCs can be visualized and tracked in the embryo, thereby providing an opportunity to investigate mechanisms that control LRO development. Previous work revealed that proliferation of DFCs via mitosis is a critical step for developing a functional KV. Here, we conducted a targeted pharmacological screen to identify mechanisms that control DFC proliferation. Small molecule inhibitors of the sarcoplasmic/endoplasmic reticulum Ca^2+^-ATPase (SERCA) were found to reduce DFC mitosis. The SERCA pump is involved in regulating intracellular calcium ion (Ca^2+^) concentration. To visualize Ca^2+^ in living embryos, we generated transgenic zebrafish using the fluorescent Ca^2+^ biosensor GCaMP6f. Live imaging identified dynamic cytoplasmic Ca^2+^ transients (“flux”) that occur unambiguously in DFCs. In addition, we report Ca^2+^ flux events that occur in the nucleus of DFCs. Nuclear Ca^2+^ flux occurred in DFCs that were about to undergo mitosis. We find that SERCA inhibitor treatments during DFC proliferation stages alters Ca^2+^ dynamics, reduces the number of ciliated cells in KV, and alters embryo laterality. Mechanistically, SERCA inhibitor treatments eliminated both cytoplasmic and nuclear Ca^2+^ flux events, and reduced progression of DFCs through the S/G2 phases of the cell cycle. These results identify SERCA-mediated Ca^2+^ signaling as a mitotic regulator of the precursor cells that give rise to the ciliated LRO.

## Introduction

Defects in establishing typical organ left-right (LR) asymmetry, known as *situs solitus*, during embryonic development can result in a mirror-image reversal of laterality, called *situs inversus totalis*, or a randomization of laterality called *situs ambiguous* (Aylsworth, 2001). *Situs inversus totalis* is associated with a low incidence of clinical consequences because the relative positioning of organs is maintained despite being in a reversed orientation. In contrast, *situs ambiguous* results in heterotaxy syndrome (HTX), which is associated with a broad spectrum of birth defects that affect the cardiovascular and gastrointestinal systems (Soofi et al., 2021). HTX-associated heart defects are variable and can be quite severe, leading to a high frequency of morbidity and mortality (Buca et al., 2018). In the 1970s, human laterality defects were linked to defects in motile cilia (Afzelius, 1976). Motile cilia are microtubule-based hair-like projections from cells that beat in a coordinated fashion to generate directional fluid flows in diverse structures that include airways, brain ventricles, and fallopian tubes (Amack, 2022). Work over the last 2 decades has identified and characterized transient structures in vertebrate embryos containing motile cilia that create asymmetric fluid flows that establish the LR body axis (Nonaka et al., 1998; Essner et al., 2002; Blum et al., 2009; Dasgupta and Amack, 2016; Hamada, 2020; Little and Norris, 2021). These ciliated structures—which include the ventral node/posterior notochordal plate in mouse and rabbit (Nonaka et al., 1998; Okada et al., 2005; Blum et al., 2007), the gastrocoel roof plate in frog (Schweickert et al., 2007), and the Kupffer’s vesicle in fish (Amack and Yost, 2004; Essner et al., 2005; Kramer-Zucker et al., 2005; Okada et al., 2005)—are now referred to as the left-right organizer (LRO) of the embryo. In these animal models, disrupting the formation or function of the ciliated LRO recapitulates laterality defects found in patients. Recent work has focused on understanding the cellular and molecular mechanisms by which the LRO breaks LR symmetry during early stages of embryogenesis.

In the mouse embryo, motile cilia generate a right-to-left fluid flow in the pit-shaped LRO that is required to establish asymmetric (left-sided) Nodal expression in the lateral plate mesoderm (Nonaka et al., 1998). Nodal, a secreted TGFβ signaling molecule, binds its receptor to activate target genes, including the transcription factor Pitx2, to mediate LR asymmetric morphogenesis of cardiovascular and gastrointestinal organs (Shiratori and Hamada, 2014). Mechanistically, motile cilia-driven fluid flows induce asymmetric Ca^2+^ signals on the left side of the LRO by activating immotile mechanosensory cilia (Mizuno et al., 2020; Katoh et al., 2023) and/or transporting signaling vesicles (Tanaka et al., 2005; Tanaka et al., 2023). These asymmetric Ca^2+^ signals trigger degradation of mRNA of the Nodal antagonist Dand5. Reduced Dand5 expression on the left side of the LRO then allows asymmetric Nodal signaling in left lateral plate mesoderm and subsequent lateralization of internal organs. A similar motile cilia-generated fluid flow- > asymmetric Ca^2+^ flux- > left-sided Nodal signaling- > LR asymmetric organ morphogenesis pathway has been characterized in zebrafish (Yuan et al., 2015; Djenoune et al., 2023). While it is established that the function of the LRO is to determine the LR axis, the pathways, mechanisms, and cell behaviors that control the formation of the LRO remain poorly understood.

**FIGURE 1:**
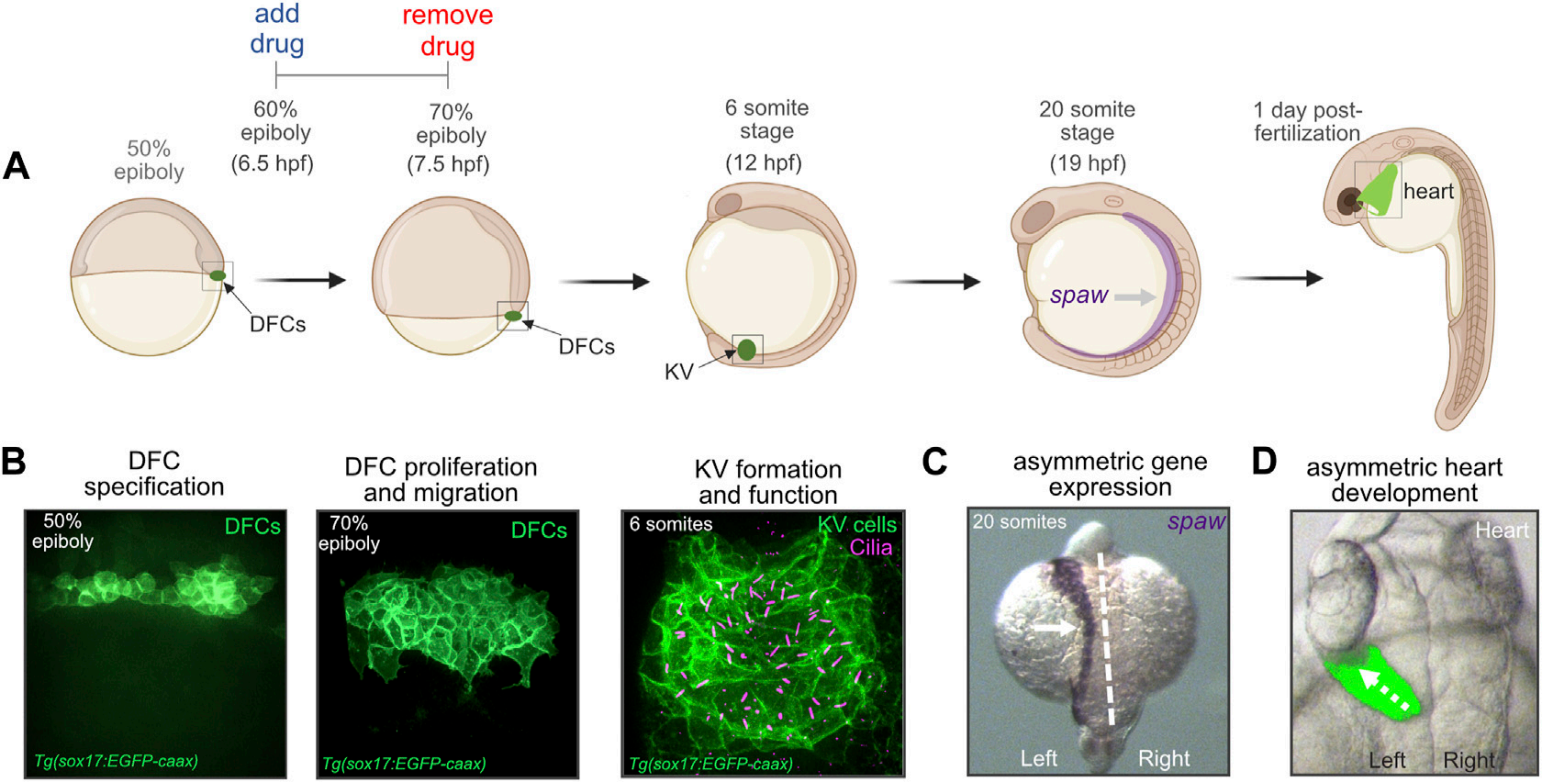
Dorsal forerunner cells give rise to the ciliated Kupffer’s vesicle that functions as the left-right organizer in the zebrafish embryo. **(A)** Diagram of early zebrafish embryo development. Dorsal forerunner cells (DFCs) are specified at the 50% epiboly stage and then form Kupffer’s vesicle (KV) by the 6 somite stage at 12 h post-fertilization (hpf). Cilia-generated fluid flow inside KV directs asymmetric expression of *spaw* and downstream asymmetric heart development. For all pharmacological treatments herein, drugs were added to embryos at the 60% epiboly stage (6.5 hpf) and removed at 70% epiboly (7.5 hpf). Created using BioRender.com. **(B)** Confocal microscopy images of DFCs and the ciliated KV. DFC/KV cells are labeled with membrane-localized EGFP expression in *Tg(sox17:EGFP-caax)* embryos. KV cilia are labeled with anti-acetylated tubulin antibodies. **(C)** RNA *in situ* hybridization of *spaw* expression in left lateral plate mesoderm (arrow) in a wild-type embryo. Dashed line marks the embryo midline. **(D)** Left-sided jogging (dashed arrow) of the heart visualized by EGFP expression driven by a *myl7* promoter in cardiomyocytes in a wild-type embryo.

In contrast to other vertebrates, the precursor cells that give rise to the LRO have been identified in the zebrafish embryo (Cooper and D'Amico, 1996; Melby et al., 1996; Warga and Kane, 2018). These precursor cells—known as dorsal forerunner cells (DFCs)—undergo specific developmental steps to form the ciliated epithelial structure called Kupffer’s vesicle (KV) that functions as the LRO in the embryo (Amack et al., 2007; Oteiza et al., 2008; Forrest et al., 2022) (Figures 1A, B). First, approximately 25 DFCs are specified in the dorsal region of the embryo at the 50% epiboly stage that occurs ∼5 h post-fertilization (hpf). Next, DFCs move posteriorly and proliferate to generate approximately 50 cells that will give rise to KV. At the tailbud stage (∼10 hpf), DFCs undergo a mesenchymal-to-epithelial transition and form a rosette-like structure. Finally, each epithelial KV cell elaborates a cilium that projects into a fluid-filled lumen that expands from the center of the rosette. Motile KV cilia create a directional fluid flow inside the KV lumen that triggers left-sided expression of a Nodal-related gene called *southpaw* (*spaw*) in lateral plate mesoderm (Long et al., 2003) (Figures 1A, C). Asymmetric organogenesis in zebrafish includes the leftward migration or “jogging” of the heart tube as it elongates (Chen et al., 1997) at 1 day post-fertilization (dpf) (Figures 1A, D), and the process of rightward “looping” at 2 dpf that is highly conserved across vertebrates (Desgrange et al., 2018). Thus, DFC/KV cells in the zebrafish embryo provides a unique system to investigate mechanisms that control LRO development and organ laterality.

Previous work indicates that proliferation of DFCs via mitotic cell division is a critical step for KV formation and function (Kim et al., 2011; Zhang et al., 2012; Arrington et al., 2013; Gokey et al., 2015; Liu et al., 2019; Rathbun et al., 2020). The proliferation of DFCs peaks during epiboly stages and then decreases as mesenchymal DFCs transition into ciliated epithelial KV cells (Gokey et al., 2015; Liu et al., 2019). Manipulations that reduce or disrupt mitosis of DFCs can alter KV formation and result in organ laterality defects. However, molecular regulators of DFC proliferation remain largely unknown. To identify new pathways that regulate the mitotic rate of DFCs we conducted a targeted *in vivo* pharmacological screen. Here, we report results from this screen that identify the sarcoplasmic/endoplasmic reticulum Ca^2+^-ATPase (SERCA) as a regulator of DFC mitosis. SERCA proteins modulate Ca^2+^ signaling in cells by pumping Ca^2+^ out of the cytoplasm and into compartments that function as Ca^2+^ stores. Ca^2+^ signaling has been implicated in LRO formation. In zebrafish, SERCA-mediated Ca^2+^ signaling has been linked to DFC clustering, gene expression, and cilia formation (Kreiling et al., 2008; Schneider et al., 2008). To investigate dynamic changes in Ca^2+^ concentration (Ca^2+^ flux) in real-time, we developed a new transgenic Ca^2+^ biosensor zebrafish strain that allows unambiguous visualization of Ca^2+^ fluxes that occur in DFCs. Live imaging revealed cytoplasmic DFC Ca^2+^ flux events and, for the first time, identified a nuclear Ca^2+^ flux in DFCs that precedes mitosis. We uncover that SERCA inhibitor treatments disrupt both cytoplasmic and nuclear Ca^2+^ flux in DFCs, and reduce progression of DFCs through the S/G2 phases of the cell cycle. These findings reveal a new function Ca^2+^ signaling in regulating proliferation during LRO development and identify novel nuclear Ca^2+^ signaling events in LRO precursors that provide a real-time marker for mitosis.

## Results

### A targeted pharmacological screen identifies Ca^2+^ signaling as a regulator of DFC proliferation

To identify pathways that mediate DFC proliferation, we conducted a small-scale pharmacological screen to target signaling pathways previously implicated in DFC/KV development. These included Wnt, Bmp, FGF, Ca^2+^, Nodal, Notch, SHH, and mTOR (Supplementary Table S1). To disrupt these pathways, we used small molecules at doses that have been successfully used in zebrafish embryos (Supplementary Table S1). Previous work indicates the proliferation rate of DFCs peaks between the 60%–70% epiboly stages that occur between 6–8 hpf (Gokey et al., 2015). We therefore focused our analyses on these stages of DFC development. For the screen, we used *Tg(sox17:EGFP-CAAX; myl7:EGFP)* transgenic embryos that express membrane-localized EGFP in the DFC/KV cell lineage driven by a *sox17* promoter, and EGFP in the heart driven by a *myl7* promoter (Dasgupta et al., 2018). These embryos, referred to here as *Tg(sox17:EGFP-caax)*, allow analysis of EGFP labeled DFCs during epiboly stages, KV cells during somite stages, and heart laterality between 1–2 dpf (Figures 1B, D).

**FIGURE 2:**
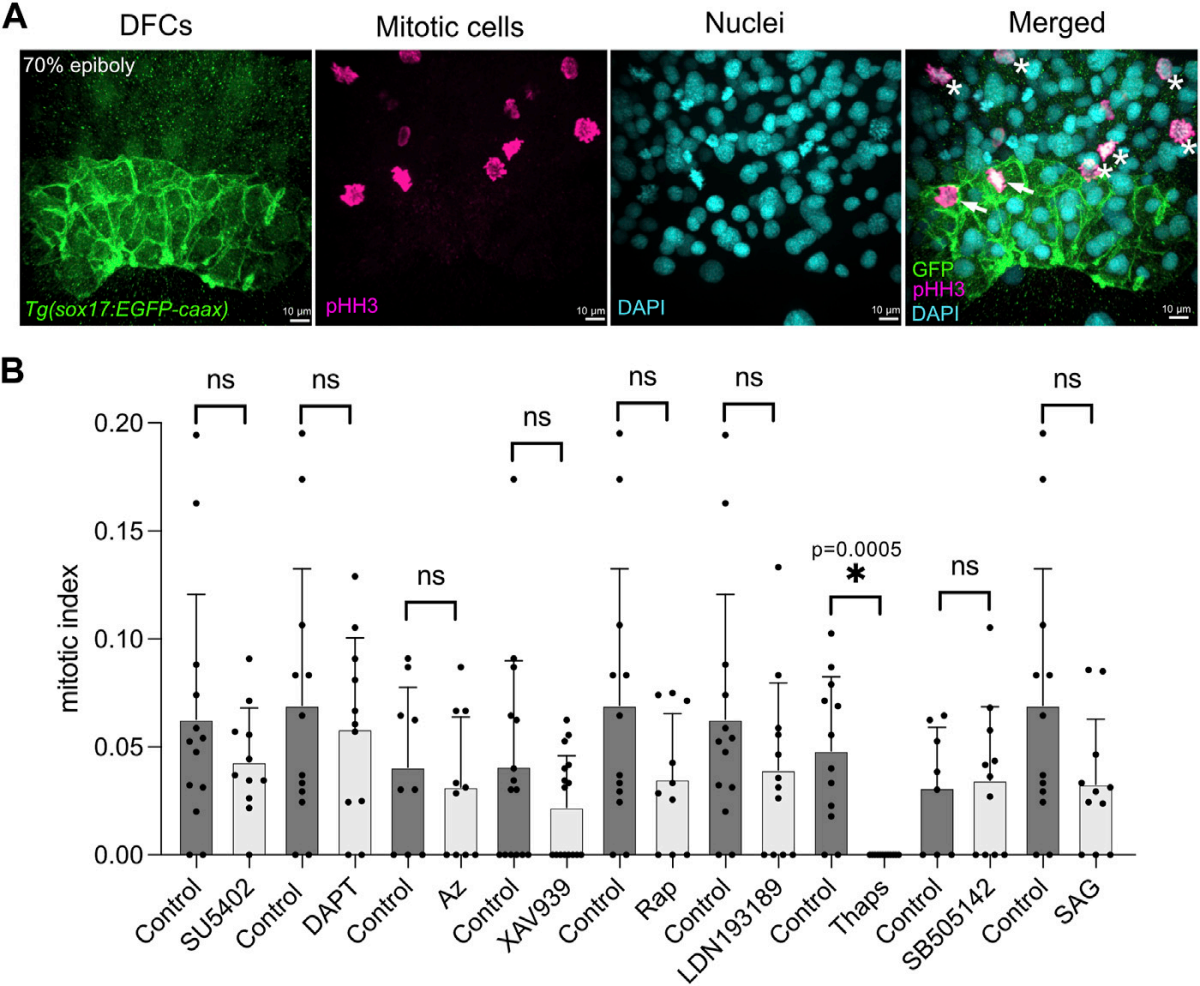
A targeted pharmacological screen to identify pathways that mediate DFC proliferation. **(A)** To quantify DFC proliferation rate, *Tg*(*sox17:EGFP-caax*) embryos were fixed at the 70% epiboly stage. Antibodies that recognize EGFP were used to label DFCs (green), and phosphorylated Histone H3 (pHH3) antibodies were used to identify cells in mitosis (magenta). DAPI staining (cyan) was used to mark all nuclei. In the merged image, arrows point out mitotic DFCs and asterisks point out mitotic neighboring (non-DFC) cells. **(B)** Results from the pharmacological screen. See Supplementary Table S1 for drug targets. The number of pHH3-positive DFCs was used to calculate a mitotic index (the percentage of DFCs in mitosis). Bar graphs indicate average values and error bars represent one standard deviation. Each circle on the graphs represents results from an individual embryo. The average mitotic index of DFCs in drug treated embryos was compared to control (DMSO treated) embryos from the same experimental group. An unpaired two-tailed *t*-test with Welch’s correction was used for statistical analysis. See Supplementary Table S2 for n values and *p* values. * = significant difference; ns = not significant.

Starting at the 60% epiboly stage, after DFCs have been specified (Oteiza et al., 2008; Warga and Kane, 2018), *Tg*(*sox17:EGFP-caax*) embryos were soaked in embryo medium containing either a specific small molecule drug or 1% dimethyl sulfoxide (DMSO) as a vehicle negative control. After incubation for 60 min at 28.5°, the treatment was removed (Figure 1A) and embryos were washed with fresh embryo medium. To analyze DFC proliferation, embryos were fixed at 70% epiboly stage and antibodies that recognize EGFP were used to label DFCs and antibodies that detect phosphorylated Histone H3 (pHH3) were used to identify cells undergoing chromatin condensation during mitosis (Hans and Dimitrov, 2001) (Figure 2A). Quantification of the number of pHH3-positive DFCs was used to calculate a mitotic index (e.g., the percentage of DFCs in mitosis) as we’ve previously described (Gokey et al., 2015). For each treatment, we analyzed on average 400 DFCs in 12 embryos from at least two independent experiments (see Supplementary Table S2). The mitotic index of DFCs in DMSO control embryos at the 70% epiboly stage was consistent with wild-type embryos in previous reports (Gokey et al., 2015; Rathbun et al., 2020); although we observed some experiment-to-experiment variability (the average mitotic index ranged between 3.1% and 6.9%) (Figure 2B; Supplementary Table S2). The average mitotic index of DFCs in drug treated embryos was compared to control DMSO treated embryos from the same experimental group (Figure 2B; Supplementary Table S2). This analysis revealed that embryos treated with small molecules targeting the FGF (SU5402), Wnt (XAV939), mTOR (Rapamycin), BMP (LDN193189), and SHH (SAG) pathways reduced the DFC mitotic index relative to controls, but these reductions were not statistically significance (Figure 2B; Supplementary Table S2). In contrast, embryos treated with thapsigargin (Thaps), a small molecule targeting the sarcoplasmic/endoplasmic reticulum Ca^2+^-ATPase (SERCA) that regulates intracellular Ca^2+^ levels (Lytton et al., 1991), eliminated pHH3 staining in DFCs (Figure 2B; Supplementary Table S2). These results implicated SERCA-mediated Ca^2+^signaling in DFC proliferation.

### SERCA inhibitor treatments during epiboly alter proliferation of DFCs, the number of ciliated KV cells, and left-right patterning of the embryo

**FIGURE 3:**
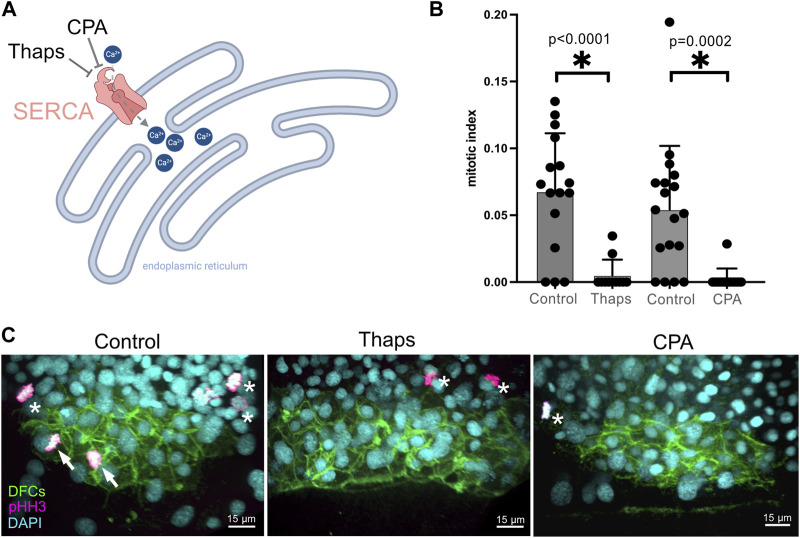
**V**alidation of screen results that identify Ca2+ signaling as a regulator of DFC proliferation. **(A)** Diagram of SERCA function to pump Ca^2+^ from the cytoplasm into endoplasmic reticulum stores, and the action of the small molecules thapsigargin (Thaps) and cyclopiazonic acid (CPA) to inhibit SERCA. Created using BioRender.com. **(B)** Treating embryos with 1 μM Thaps or 100 μM CPA significantly reduced the mitotic index of DFCs as compared to negative control DMSO treated embryos. Bar graphs indicate average values and error bars represent one standard deviation. Each circle on the graphs represents results from an individual embryo. An unpaired two-tailed *t*-test with Welch’s correction was used for statistical analysis. * = significant difference. n = number of embryos analyzed. **(C)** Representative merged images of pHH3 staining in *Tg*(*sox17:EGFP-caax*) embryos treated with 1% DMSO (negative control), 1 μM Thaps, or 100 μM CPA. Arrows point out mitotic DFCs and asterisks point out mitotic neighboring (non-DFC) cells.

Based on the results of our pharmacological screen, we focused on characterizing the role of SERCA activity during DFC proliferation and formation of the ciliated KV. The SERCA pump uses ATP to transport Ca^2+^ from the cytoplasm to the endoplasmic reticulum or sarcoplasmic reticulum (Primeau et al., 2018), or the nuclear envelope (Lanini et al., 1992; Collado-Hilly et al., 2010) (Figure 3A). Blocking SERCA activity has been found to initially increase cytoplasmic Ca^2+^ levels and ultimately deplete Ca^2+^ stores thereby altering Ca^2+^ signaling dynamics. RNA sequencing of isolated DFCs (our unpublished data) indicates that two out of the three genes (*atp6a2b* and *atpa2a*, but not *atp2a1*) that encode SERCA pumps are expressed in these cells. To avoid potential genetic compensation, pleiotropic effects, and/or off-target effects that can complicate gene knockdown approaches, we chose to interfere with SERCA-mediated Ca^2+^signaling using pharmacological inhibitors that offer tunable doses and temporal control of treatments. To validate results from the screen, we 1) repeated the thapsigargin treatments and 2) treated embryos with a second SERCA inhibitor called cyclopiazonic acid (CPA) that has been effectively used in zebrafish embryos (Kreiling et al., 2008; Schneider et al., 2008). Consistent with results from the screen, new 1 μM thapsigargin treatments at 60% epiboly for 60 min significantly reduced the DFC mitotic index at the 70% epiboly stage (Figures 3B, C; Supplementary Table S3). Similarly, treating embryos with 100 μM CPA at 60% epiboly significantly reduced the number of mitotic DFCs (Figures 3B, C; Supplementary Table S3). Importantly, we did not observe any developmental delay in treated embryos relative to DMSO controls. To test the specificity of SERCA inhibition on DFC proliferation, we quantified the mitotic index of neighboring dorsal margin cells adjacent to DFCs. In contrast to the reduction of proliferating DFCs in thapsigargin treated embryos, there was no significant difference in the mitotic index in neighboring non-DFC cells as compared to DMSO treated controls (Supplementary Figure S1). These results indicate that SERCA-mediated Ca^2+^signaling during epiboly is a regulator of DFC proliferation.

**FIGURE 4:**
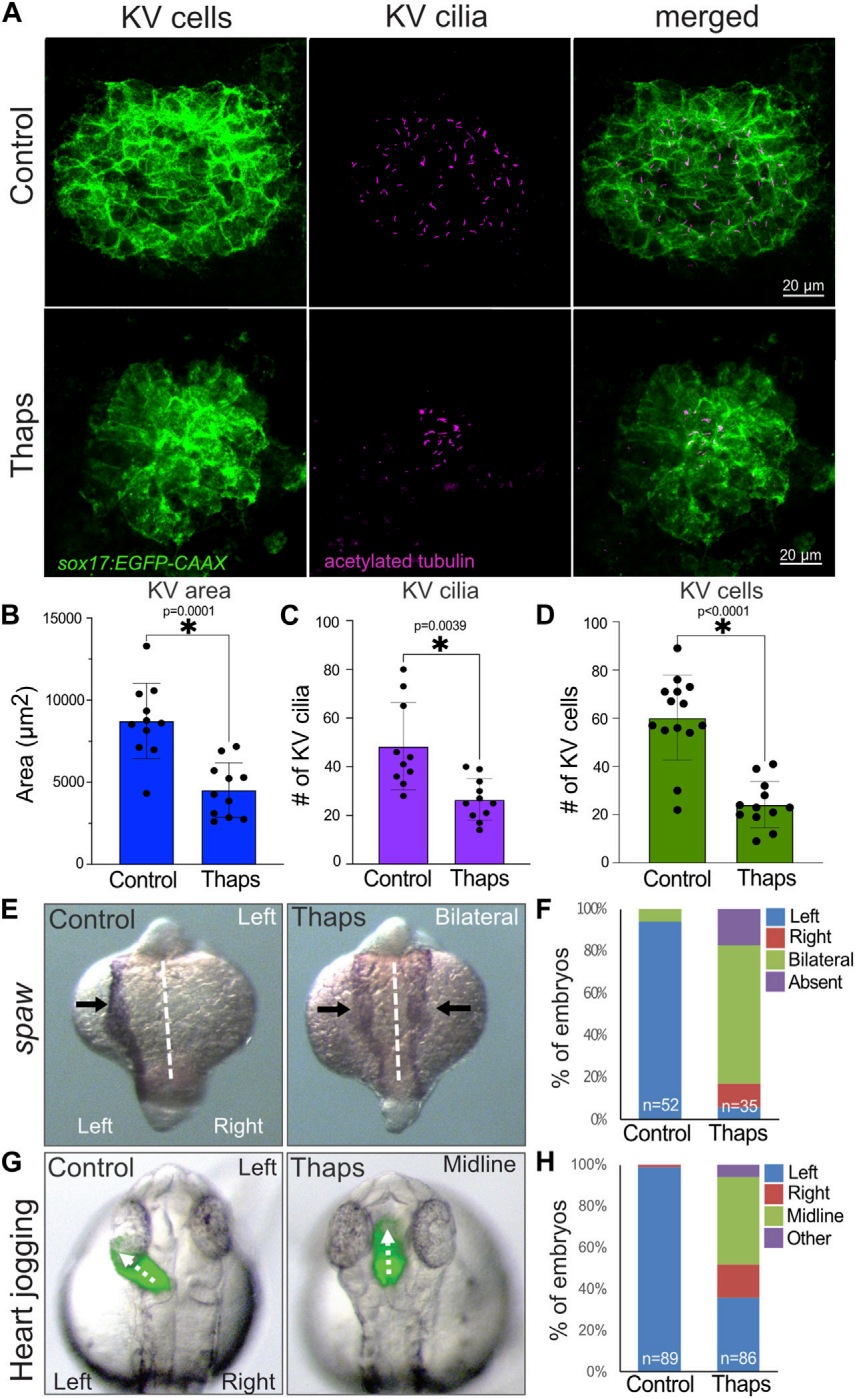
SERCA inhibitor treatments during epiboly reduce the number of ciliated KV cells and disrupt left-right patterning of the embryo. **(A)** Representative images of the ciliated KV in *Tg*(*sox17:EGFP-caax*) embryos treated with 1% DMSO (control) or 1 μM Thapsigargin (Thaps). KV cells are labeled with membrane-localized EGFP expression (green), and KV cilia are labeled with anti-acetylated tubulin antibodies (magenta). **(B–D)** Treating embryos with 1 μM Thapsigargin during epiboly stages reduced the area of KV **(B)**, the number of cilia in KV **(C)**, and the number of cells in KV **(D)** relative to DMSO treated controls. Bar graphs indicate average values and error bars represent one standard deviation. Each circle on the graphs represents results from an individual embryo. An unpaired two-tailed *t*-test with Welch’s correction was used for statistical analysis. * = significant difference. **(E)** Representative images of normal left-sided *spaw* expression (arrows) in a DMSO control embryo and bilateral expression in a thapsigargin treated embryo. **(F)** Most DMSO control embryos had left-sided *spaw* expression, whereas *spaw* was largely bilateral, and in some cases absent or right-sided in thapsigargin treated embryos. **(G)** Representative images of normal leftward heart jogging (dashed arrow) in a DMSO treated control embryo and midline jogging in a thapsigargin treated embryo. The heart tube was labeled by EGFP expression in cardiomyocytes. **(H)** Leftward heart jogging was observed in most control embryos. In contrast, the heart often jogged along the midline or to the right in thapsigargin treated embryos. *n* = number of embryos analyzed.

Since SERCA inhibitor treatments reduced the DFC mitotic index during epiboly stages, we predicted this would result in a reduced number of ciliated KV cells during subsequent somite stages. Motile cilia protrude from the apical surface of epithelial KV cells into the fluid filled KV lumen. KV cilia generate (Essner et al., 2005; Kramer-Zucker et al., 2005) and likely sense (Yuan et al., 2015; Djenoune et al., 2023) a directional fluid flow that is critical for LR asymmetric signaling and organ patterning. Ciliated KV cells were visualized using *Tg*(*sox17:EGFP-caax*) embryos that mark KV cells and anti-acetylated tubulin antibodies that label KV cilia. Embryos treated with 1% DMSO or 1 μM thapsigargin at the 60% epiboly stage for 60 min were washed and allowed to develop to the six to eight somite stages to analyze KV. KVs in thapsigargin treated embryos were significantly smaller than KVs in DMSO treated controls (Figures 4A, B). In addition, the number of KV cilia was significantly reduced in embryos treated with thapsigargin relative to controls (Figures 4A, C). We next quantified the number of KV cells at the 6-somite stage as we’ve previously described (Tay et al., 2013). This analysis indicated the number of KV cells was significantly reduced in thapsigargin treated embryos as compared to control DMSO treated embryos (Figures 4A, D). Since both the number of KV cilia and the number of KV cells were reduced in thapsigargin treated embryos, we interpret these results to indicate that interfering with SERCA-mediated Ca^2+^signaling during epiboly stages does not impact KV ciliogenesis, but rather reduces the number of ciliated cells in KV.

Since thapsigargin treatments during epiboly stages inhibit DFC mitosis and reduce the number of ciliated KV cells, we next asked whether these treatments alter LR patterning of the embryo. In previous work, treating embryos with thapsigargin during epiboly using either higher doses (2.5 μM) (Schneider et al., 2008) or longer exposures (2 h) (Kreiling et al., 2008) resulted in laterality defects. To assess laterality in embryos treated with 1 μM thapsigargin at 60% epiboly for 60 min, we first analyzed expression of the nodal-related gene *spaw* in lateral plate mesoderm (Figure 4E). Normal left-sided expression of *spaw* was detected in most DMSO control embryos, whereas *spaw* was bilateral, right-sided, or absent in thapsigargin treated embryos (Figure 4F). Consistent with *spaw* defects in thapsigargin treated embryos, direction of heart jogging was disrupted in these embryos at 1 dpf (Figure 4G). Asymmetric left-sided heart jogging was observed in control embryos, whereas the heart often remained along the midline or jogged to the right in thapsigargin treated embryos (Figure 4H). At 2 dpf, DMSO and thapsigargin treated embryos had similar gross morphologies (Supplementary Figures S2A, B). However, the direction of heart looping was often along the midline or reversed in thapsigargin treated embryos (Supplementary Figures S2C–F). Although these outcomes are reminiscent of laterality defects induced by other treatments that reduce DFC proliferation (Arrington et al., 2013; Gokey et al., 2015; Liu et al., 2019), the disruption of LR asymmetry in thapsigargin treated embryos may be due to pleiotropic effects. Thus, it is not possible to conclude that laterality defects in thapsigargin treated embryos are directly due to small KVs in these embryos. However, the correlation of laterality defects with KV defects is consistent with a model in which SERCA-mediated Ca^2+^signaling during epiboly mediates DFC proliferation that regulates the number of ciliated KV cells, which in turn, impacts LR patterning of the embryo.

### Inhibiting SERCA alters cell cycle progression of DFCs

**FIGURE 5:**
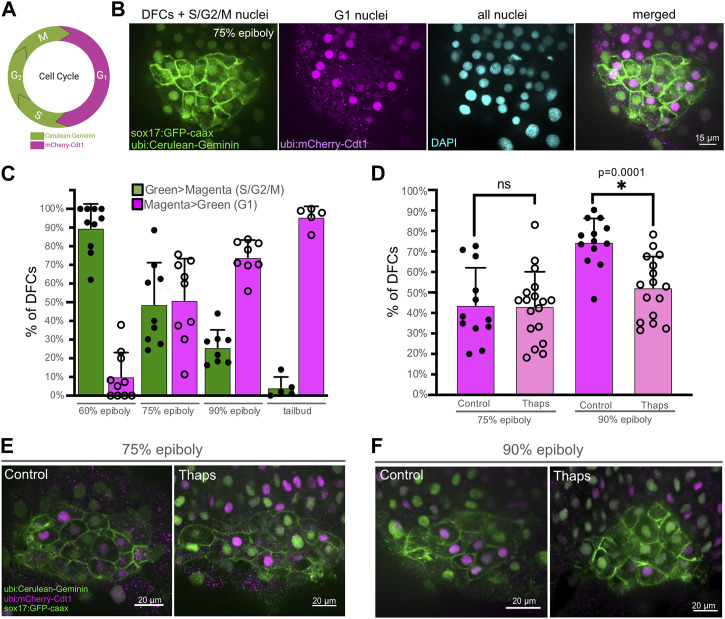
SERCA activity meditates progression of DFCs through the S/G2 phases of the cell cycle. **(A)** Diagram of transgene expression during the cell cycle in Dual-FUCCI transgenic zebrafish. Created using BioRender.com. **(B)** Representative image of a double *Tg*(*Dual-FUCCI*); *Tg*(*sox17:EGFP-caax*) embryo at the 75% epiboly stage. DFC cell membranes are labeled by *Tg*(*sox17:EGFP-caax*) expression (green), and fluorescent antibody staining was used to detect mCherry-Cdt1 (magenta) and cerulean-Geminin (green). DAPI staining (cyan) was used to mark all nuclei. **(C)** Analysis of cell cycle status of DFCs in wild-type *Tg(Dual-FUCCI)*; *Tg*(*sox17:EGFP-caax*) embryos at different developmental stages. Bar graphs indicate average values and error bars represent one standard deviation. Each circle on the graphs represents results from an individual embryo. **(D)** Quantification of the percentage of DFCs in G1 (magenta > green) in *Tg(Dual-FUCCI)*; *Tg*(*sox17:EGFP-caax*) embryos treated with 1% DMSO (control) or 1 μM thapsigargin at 60% epiboly for 60 min and then fixed at 75% epiboly or 90% epiboly. An unpaired two-tailed *t*-test with Welch’s correction was used for statistical analysis. * = significant difference; ns = not significant. **(E, F)** Representative images of control or thapsigargin treated *Tg(Dual-FUCCI)*; *Tg*(*sox17:EGFP-caax*) embryos at the 75% epiboly **(E)** or 90% epiboly **(F)** stage.

To investigate the cellular mechanism by which SERCA inhibitor treatments reduce DFC proliferation, we developed methods using *Tg*(*Dual-FUCCI*) transgenic embryos to analyze the cell cycle of DFCs. The cell cycle consists of four phases: gap/growth 1 (G1), DNA synthesis (S), gap/growth 2 (G2), and mitosis (M). The previously described Dual-FUCCI (Fluorescent Ubiquitination Cell-Cycle Indicator) transgenic strain ubiquitously expresses an mCherry-Cdt1 fusion protein that accumulates in the nucleus during G1 phase, and a cerulean-Geminin fusion protein that accumulates in the nucleus during S, G2, and early M phases (Bouldin et al., 2014) (Figure 5A). Generating double *Tg*(*Dual-FUCCI*); *Tg*(*sox17:EGFP-caax*) embryos allowed us to visualize of the cell cycle status of each individual DFC marked by the *Tg*(*sox17:EGFP-caax*) transgene (Figure 5B). We first characterized cell cycle status of DFCs in wild-type embryos at different developmental stages. Because transgene expression was low during epiboly stages, we used antibodies to detect mCherry-Cdt1 (pseudo-colored magenta) and cerulean-Geminin (pseudo-colored green). At the 60% epiboly stage, an average of 90% ± 12% of DFCs (*n* = 10 embryos) showed strong nuclear fluorescence of the cerulean-Geminin protein indicating the cells were in the S/G2/M phases, whereas 10% ± 12% of the DFCs had strong mCherry-Cdt1 expression indicating G1 phase (Figure 5C). During subsequent stages (75% epiboly, 90% epiboly, and tailbud) the percentage of DFCs with cerulean-Geminin protein expression decreased, and the percentage of DFCs with mCherry-Cdt1 expression increased (Figure 5C). These results indicate the number of DFCs in S/G2/M phases decrease during later epiboly stages. This is consistent with previous work using other cell cycle markers that indicates the proliferation of mesenchymal DFCs peaks ∼60%–70% epiboly and then decreases as DFCs differentiate into ciliated epithelial KV cells (Gokey et al., 2015; Liu et al., 2019).

Next, we used *Tg*(*Dual-FUCCI*); *Tg*(*sox17:EGFP-caax*) embryos to determine how SERCA inhibitor treatments impact the cell cycle of DFCs. Since our pHH3 analysis indicates thapsigargin blocks DFCs from entering mitosis (Figure 3), we reasoned there were two plausible mechanisms. First, thapsigargin may cause DFCs to accumulate in G1. Work in human cell cultures has found that inhibiting SERCA attenuates the G1- > S transition (Simon and Moran, 2001). If this was the case in DFCs, we would expect to find more mCherry-Cdt1 positive (G1) DFCs in thapsigargin treated embryos relative to controls. A second possibility is that thapsigargin treated DFCs stall and fail to progress through S/G2. In this case, we would see fewer mCherry-Cdt1 positive (G1) DFCs in thapsigargin treated embryos. To address this, *Tg*(*Dual-FUCCI*); *Tg*(*sox17:EGFP-caax*) embryos were treated with 1 μM thapsigargin for 60 min starting at the 60% epiboly stage and compared to 1% DMSO controls. Following treatments, embryos were fixed at either the 75% epiboly or 90% epiboly stage for antibody staining of mCherry-Cdt1 (G1) and cerulean-Geminin (S/G2/M). Similar to wild-type embryos, the percentage of DFCs in G1 increased between 75% and 90% epiboly in DMSO control embryos (Figures 5D–F; Supplementary Table S4). In contrast, the percentage of DFCs in G1 remained similar between 75% and 90% epiboly in thapsigargin treated embryos (Figures 5D–F; Supplementary Table S4). There was no significant difference in G1 DFCs between DMSO and thapsigargin treated embryos at 75% epiboly. However, at 90% epiboly, the percentage of DFCs in G1 was significantly reduced by thapsigargin treatments (Figure 5D; Supplementary Table S4). Taken together with results that entry into M phase (mitotic index) is significantly reduced in thapsigargin treated embryos (Figure 3), these results indicate that inhibiting SERCA stalls DFCs in the S/G2 phases of the cell cycle.

### Visualization of SERCA-dependent Ca^2+^ flux in DFCs

We next wanted to investigate the effect of our thapsigargin treatments on Ca^2+^ flux in live embryos. To visualize Ca^2+^ dynamics with high temporal resolution, we generated a stable transgenic *Tg*(*act2b:GCaMP6f*) zebrafish strain that ubiquitously expresses the genetically encoded Ca^2+^ sensor GCaMP6f, which increases fluorescence upon Ca^2+^ binding with fast (f) dynamics (Chen et al., 2013). To determine whether *Tg*(*act2b:GCaMP6f*) zebrafish function as a robust Ca^2+^ reporter strain, we asked whether we could visualize fluctuations of GCaMP6f fluorescence intensity in structures that are known to require dynamic Ca^2+^ signals. Using real-time confocal microscopy, we observed waves of fluorescence signal during heart contractions (Supplementary Movie S1), flashes in the olfactory pit (Supplementary Movie S2), and movement activated flux in hair cells in neuromasts of the lateral line (Supplementary Movie S3). These results indicate the *Tg*(*act2b:GCaMP6f*) strain provides a useful new Ca^2+^ reporter with fast dynamics in zebrafish.

**FIGURE 6:**
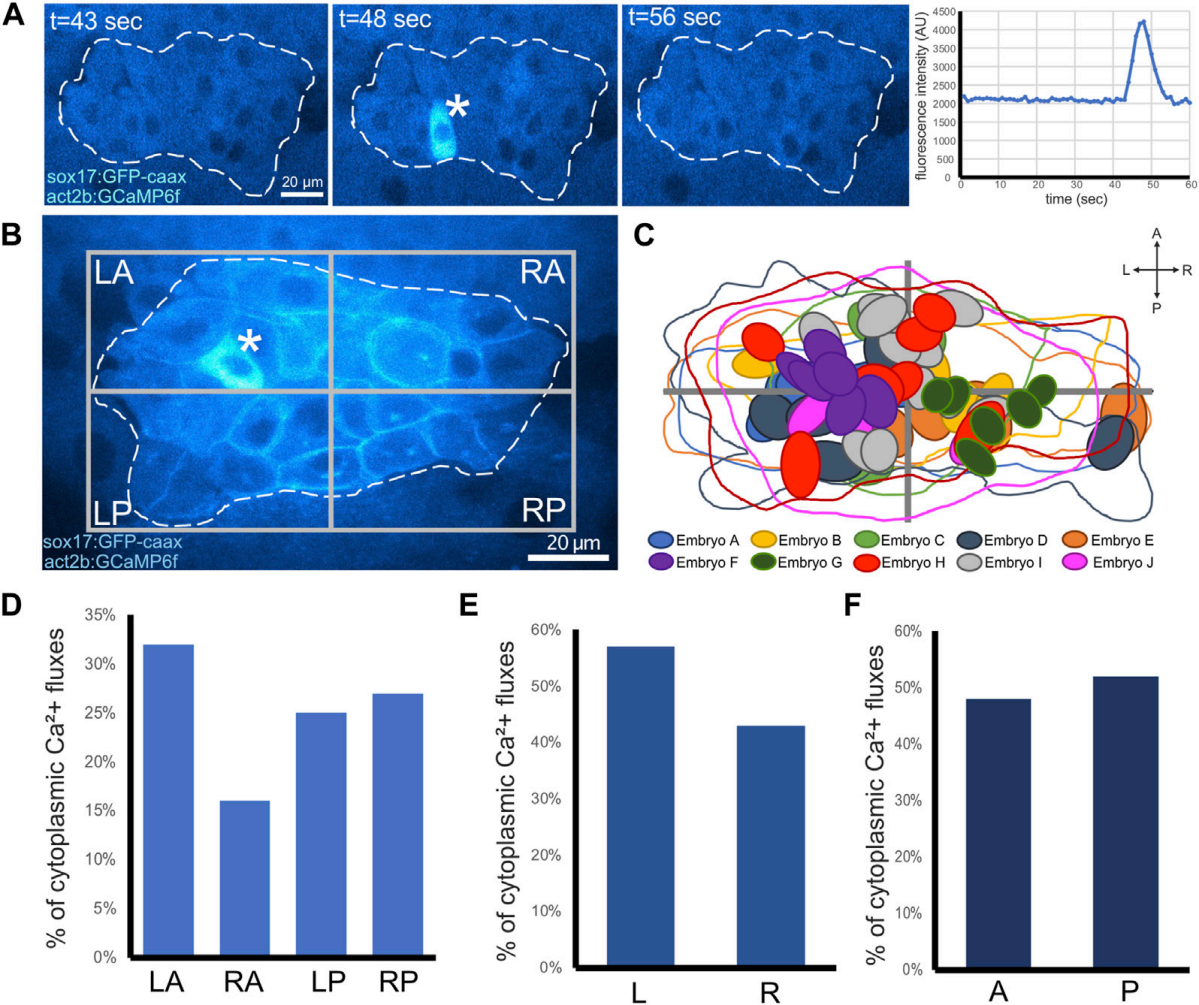
Visualization of cytoplasmic Ca2+ flux events in DFCs. **(A)** Confocal time-lapse images of a single Z-plane through DFCs in a *Tg*(*act2b:GCaMP6f*); *Tg*(*sox17:EGFP-caax*) embryo at the 60% epiboly stage. Changes in Ca^2+^ concentration (GCaMP6f fluorescence intensity) are visualized by changes in intensity of the cyan hot lookup table (FIJI software). The asterisk marks a DFC that undergoes a transient cytoplasmic Ca^2+^ flux. Dashed white line indicates the boundary of the DFC cluster. The graph plots GCaMP6f fluorescence intensity in the cytoplasm in the DFC over time. t = time in sec. AU = arbitrary units. **(B)** Image of a representative DFC cluster in a *Tg*(*act2b:GCaMP6f*); *Tg*(*sox17:EGFP-caax*) embryo. Dashed white line indicates the boundary of the DFC cluster, which is used to define left-anterior (LA), right-anterior (RA), left-posterior (LP), and right-posterior (RP) quadrants. The asterisk indicates a DFC cytoplasmic Ca^2+^ flux event. **(C)** Overlay of the DFC cluster boundaries and cytoplasmic Ca^2+^ flux events in all embryos analyzed (n = 56 events from 10 embryos) reveals spatial location of cytoplasmic Ca^2+^ fluxes in DFCs. **(D–F)** Quantification of pooled DFC cytoplasmic Ca^2+^ flux events in DFC quadrants **(D)**, along the LR axis **(E)**, and along the AP axis **(F)**.

In previous work, the Ca^2+^ sensors Oregon Green BAPTA-1 Dextran (Kreiling et al., 2008) or Fura-2 (Schneider et al., 2008) were used to visualize and measure Ca^2+^ levels in zebrafish embryos. Transient Ca^2+^ fluxes were identified in the region of DFCs and were most abundant at the 60%–70% epiboly stages (Schneider et al., 2008). More recently, transgenic zebrafish embryos ubiquitously expressing the genetically encoded Ca^2+^ indicator GCaMP6s, which increases fluorescence upon Ca^2+^ binding with slow (s) dynamics (Chen et al., 2013), were used to visualize Ca^2+^ flux activity in the dorsal region of the embryo during epiboly with higher spatial resolution (Chen et al., 2017). This study characterized Ca^2+^ fluxes in epithelial enveloping layer (EVL) cells and in smaller mesenchymal DFCs. Consistent with this report, we observed dynamic Ca^2+^ signals in our *Tg*(*act2b:GCaMP6f*) embryos during epiboly stages at the dorsal margin that included DFCs, neighboring margin cells, and overlying EVL cells. Like previous studies that lacked a specific marker for DFCs, we could only classify cells as DFCs based on morphology and location near and/or below the dorsal margin in these embryos. To explicitly identify DFCs and more precisely quantify Ca^2+^ signals that occur in DFCs, we generated double *Tg*(*act2b:GCaMP6f*); *Tg*(*sox17:EGFP-caax*) transgenic embryos. These embryos allow simultaneous detection of DFCs and visualization of Ca^2+^ dynamics at high temporal and spatial resolution using spinning disk confocal microscopy. This approach allowed us to detect and analyze transient Ca^2+^ increases that unambiguously occur in the cytoplasm of DFCs (Supplementary Movie S4; Figure 6A).

To determine the duration of transient Ca^2+^ increases, or Ca^2+^ flux events, in DFCs, we imaged *Tg*(*act2b:GCaMP6f*); *Tg*(*sox17:EGFP-caax*) embryos at single focal plane at 60% epiboly for 1 min using a 1 s interval. Intensity profiles of GCaMP6f fluorescence were plotted for individual cells to identify the start (defined as the timepoint when GCaMP6f fluorescence increases above baseline levels) and end (defined as the return to baseline) of a Ca^2+^ flux event (Figure 6A). This analysis indicated that the duration of these DFC cytoplasmic Ca^2+^ flux events ranged from 5 to 17 s and the average was 11.7 ± 3.4 s (Supplementary Table S5). We next estimated the frequency of DFC cytoplasmic Ca^2+^ transients per embryo by imaging the entire DFC cluster in *Tg*(*act2b:GCaMP6f*); *Tg*(*sox17:EGFP-caax*) embryos starting at 60% or 70% epiboly stage for longer time periods. We used a 15 s interval, which was the minimum time needed to image through all DFCs. To minimize photodamage, we imaged each embryo for 10–30 min. Since our imaging interval (15 s) was longer than the average duration of Ca^2+^ events (11.7 s), this analysis could miss some events and can therefore only provide an estimate of the frequency. We found that the number of cytoplasmic Ca^2+^ flux events in the DFC cluster was variable from embryo to embryo, but on average our analysis detected DFC cytoplasmic flux events at a normalized rate of 20.2 ± 13.6 events/hour/embryo at 60% epiboly and a similar 21.0 ± 16.0 events/hour/embryo at 70% epiboly (Supplementary Table S6). In some cases, the same DFC showed multiple cytoplasmic Ca^2+^ flux events during imaging (see Supplementary Movie S8). These results establish new methods to visualize transient Ca^2+^ signals specifically in DFCs with fast dynamics, and provide new insights into the duration, frequency, and variability of Ca^2+^ flux in DFCs.

We then asked whether there was a pattern to the cytoplasmic Ca^2+^ flux events in DFCs. We generated spatial maps to determine the relative positions that Ca^2+^ flux events occurred within DFCs in a pooled population of 10 embryos (Figures 6B, C; Supplementary Table S7). Intriguingly, in each embryo we analyzed, we observed regional clusters of DFCs that experienced Ca^2+^ flux during the imaging time window (Supplementary Figure S3). This suggests local coordination of Ca^2+^ signaling. We observed cytoplasmic flux events in all quadrants of the DFCs, with a slight bias towards the left-anterior (LA) quadrant (Figures 6C, D). In addition, we found a slight bias of Ca^2+^ flux in left-sided DFCs (Figure 6E) and very little difference along the anterior-posterior DFC axis (Figure 6F). Finally, we determined the impact of SERCA inhibitor treatments on DFC cytoplasmic Ca^2+^ flux events. Similar to wild-type embryos, DMSO treated control embryos showed DFC cytoplasmic Ca^2+^ flux events (Supplementary Movie S5) at a rate of 18.5 ± 13.2 events/hour at 70% epiboly (Supplementary Table S8). In contrast, treatments with 1 μM thapsigargin or 100 μM CPA initially increased the cytoplasmic Ca^2+^ concentration and abolished Ca^2+^ flux events in DFCs and surrounding cells (Supplementary Movie S6; Supplementary Movie S7; Supplementary Table S8). Taken together, these findings identify SERCA-dependent cytoplasmic Ca^2+^ flux events with variable frequency and location in DFCs.

### Identification of nuclear Ca^2+^ flux in DFCs

**FIGURE 7:**
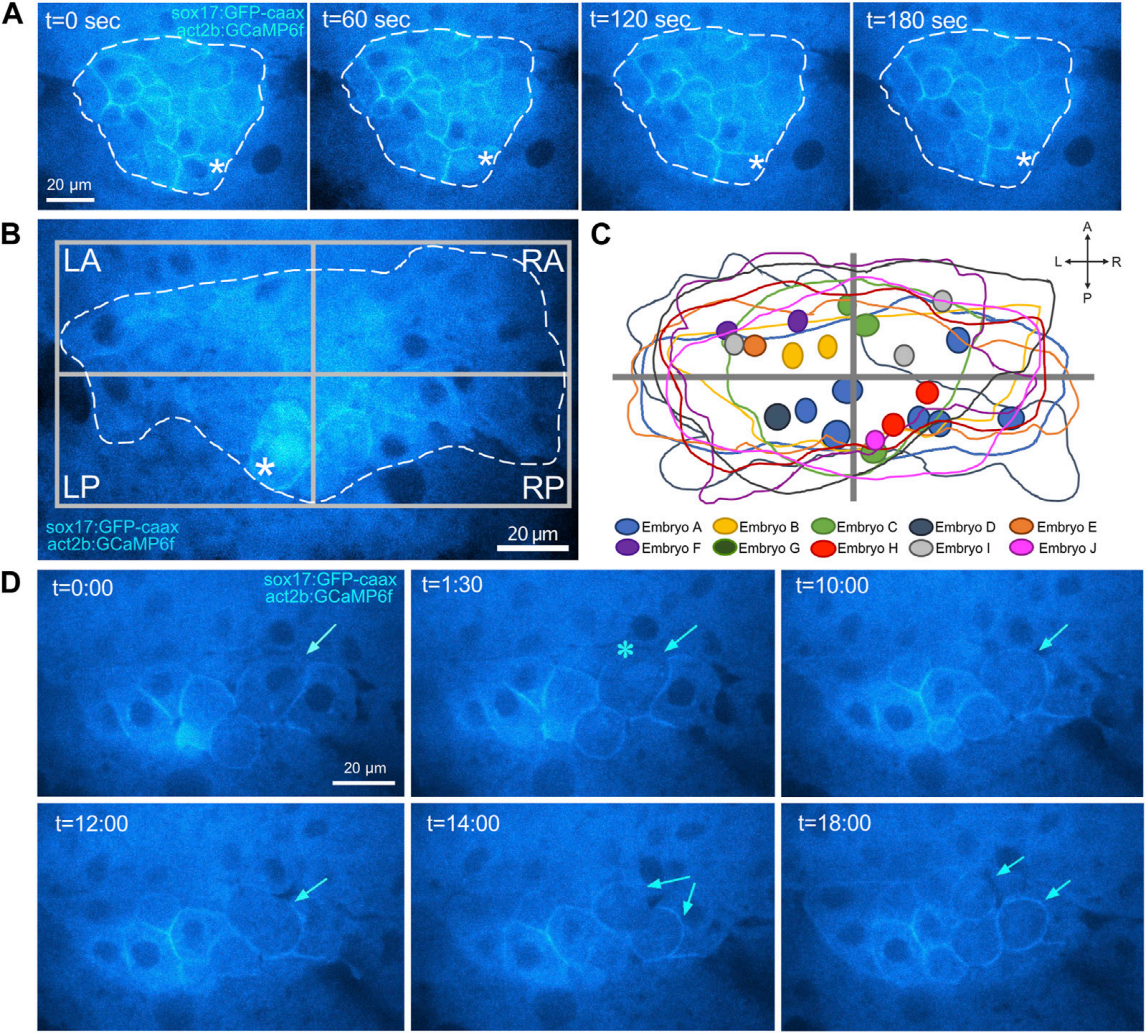
Identification of nuclear Ca2+ flux in DFCs. **(A)** Confocal images of a single Z-plane through DFCs in a *Tg*(*act2b:GCaMP6f*); *Tg*(*sox17:EGFP-caax*) embryo at the 60% epiboly stage. Changes in Ca^2+^ concentration (GCaMP6f fluorescence intensity) are visualized by changes in intensity of the cyan hot lookup table (FIJI software). The asterisks mark a transient increase in Ca^2+^ concentration in the nucleus of a DFC. Dashed white line indicates the boundary of the DFC cluster. t = time in sec. **(B)** Image of a representative DFC cluster in a *Tg*(*act2b:GCaMP6f*); *Tg*(*sox17:EGFP-caax*) embryo. Dashed white line indicates the boundary of the DFC cluster, which is used to define quadrants. The asterisk indicates a DFC nuclear Ca^2+^ flux event. **(C)** Overlay of the DFC cluster boundaries and locations of nuclear Ca^2+^ flux events in all embryos analyzed (*n* = 22 events from nine embryos). **(D)** Confocal time-lapse images of DFCs in a *Tg*(*act2b:GCaMP6f*); *Tg*(*sox17:EGFP-caax*) embryo. Following a nuclear Ca^2+^ flux event (asterisk), the DFC (arrow) rounds up and divides into two daughter cells. t = min:sec.

In addition to cytoplasmic Ca^2+^ flux events, live imaging of *Tg*(*act2b:GCaMP6f*); *Tg*(*sox17:EGFP-caax*) embryos revealed infrequent transient Ca^2+^ spikes in the nuclei of DFCs (Figure 7A). The identification of nuclear Ca^2+^ flux in DFCs is intriguing because nuclear Ca^2+^ signaling has been implicated in regulating gene expression and cell cycle in other systems (Resende et al., 2013). The frequency of nuclear Ca^2+^ flux in DFCs was again variable from embryo to embryo, but we detected an average rate of 5.9 ± 5.5 events/hour/embryo at 60% epiboly and a reduced rate of 3.0 ± 4.5 events/hour/embryo at 70% epiboly (Supplementary Table S6). Similar to cytoplasmic Ca^2+^ fluxes in DFCs, spatial mapping of nuclear Ca^2+^ signals (22 events in nine embryos) indicated a slight bias towards the left-anterior quadrant and left side and of the DFC cluster (Figures 7B, C; Supplementary Figure S4; Supplementary Table S7). Also similar to cytoplasmic signals, nuclear DFC Ca^2+^ signals were observed in DMSO treated controls but were not detected in Thapsigargin or CPA treated embryos (Supplementary Table S8). These results identify for the first time SERCA-dependent nuclear Ca^2+^ flux events in DFCs.

### Nuclear Ca^2+^ flux events provide a real-time marker for DFC mitosis

We next used time-lapse imaging to track and characterize cell behaviors of DFCs following Ca^2+^ flux events. First, we tracked DFCs that showed a cytoplasmic Ca^2+^ flux event (*n* = 10 cells from seven embryos imaged during five independent experiments). Tracking these cells for up to 20 min did not reveal any gross changes in cell morphology, behavior, or position (Supplementary Movie S8; Supplementary Table S9). We next tracked DFCs following a nuclear Ca^2+^ flux event (*n* = 10 cells from seven embryos imaged during five independent experiments). In contrast to cytoplasmic Ca^2+^ events, nuclear Ca^2+^ flux was associated with cell division of DFCs (Supplementary Movie S9; Figure 7D). Nine out of ten DFCs analyzed were found to change to a rounded morphology and then divide into two daughter DFCs within the imaging time window (Supplementary Table S9). The average time between nuclear Ca^2+^ flux and cell division was 12.2 ± 3.86 min. For the one DFC that did not divide, the imaging period ended after 10 min. We predict we would have observed this DFC divide if we had imaged long enough. To address whether nuclear Ca^2+^ flux always precedes division, we tracked DFCs that did not show nuclear Ca^2+^ flux (or cytoplasmic Ca^2+^ flux) and asked if any of these cells divided. We tracked 20 cells from five embryos from three independent experiments (for 20–40 min) and none of these cells divided (Supplementary Table S10). These experiments suggest nuclear Ca^2+^ flux precedes all DFC cell division, and thereby provides a real-time marker for DFCs that will undergo mitosis.

## Discussion

The DFC/KV cell lineage in the zebrafish embryo provides a unique window into LRO development. Previous work has identified mitotic cell division of DFCs during epiboly stages as a critical step towards building a functional KV (Gokey et al., 2015; Liu et al., 2019; Rathbun et al., 2020). Here, we report results from a small-scale targeted pharmacological screen that has identified Ca^2+^ signaling as a regulator of DFC mitosis. Due to limitations of our pharmacological screen, we cannot conclusively rule out any of the tested pathways as regulators of DFC/KV proliferation. Small molecule inhibitors of FGF, Wnt, mTOR, BMP, and SHH pathways resulted in modest reductions in DFC mitotic index (Figure 2B; Supplementary Table S2). While these reductions were not significantly different from negative controls in our screen, it is possible that different drug doses or exposure times could result in a significant impact on DFC proliferation. It also remains possible that several pathways interact to coordinate DFC proliferation, and that simultaneously inhibiting multiple pathways may result in additive or synergistic effects. While results from the screen provide a starting point for future work to address these questions, the overarching goal of the screen was to identify big effects on mitotic index of DFCs at a very specific point in development. Our finding that thapsigargin treatments suppressed the DFC mitotic index represented a significant ‘hit’ in the screen. We therefore focused on Ca^2+^ signaling during DFC proliferation and KV development.

### Multiple functions for Ca^2+^ signaling during development of LR asymmetry

SERCA controls cytosolic Ca^2+^ concentration by translocating Ca^2+^ out of the cytoplasm into subcellular Ca^2+^ stores (Figure 3A). SERCA activity can thereby regulate the dynamics of transient increases in cytosolic Ca^2+^ levels from internal stores or the external environment that serve as signals to impact cell behavior. Transient Ca^2+^ signals, or flux events, regulate a number of cellular processes, including contraction, secretion, metabolism, gene expression, and proliferation (Bootman and Bultynck, 2020). Ca^2+^ mediates these downstream effects in a context-dependent manner by interacting with an extensive array of Ca^2+^-binding proteins (Clapham, 2007). Previous work in several systems has revealed multiple roles for Ca^2+^-mediated signaling during embryonic development (Slusarski and Pelegri, 2007). As a prime example, Ca^2+^ flux has emerged as a key component of vertebrate LR patterning (Langenbacher and Chen, 2008; Norris, 2012; Yoshiba and Hamada, 2014; Blum and Vick, 2015). Asymmetric Ca^2+^ signaling has been observed on the left side of the LRO in mouse and zebrafish (McGrath et al., 2003; Sarmah et al., 2005; Tanaka et al., 2005; Jurynec et al., 2008; Francescatto et al., 2010; Takao et al., 2013), and inhibiting these signals alters LR patterning. Ca^2+^ signaling has also been implicated in LR development in frogs and birds (Raya et al., 2004; Garic-Stankovic et al., 2008; Hatayama et al., 2011). Recent work in mouse and zebrafish indicates asymmetric Ca^2+^ flux events at the LRO are generated via a mechanosensory mechanism in response to leftward fluid flows created by motile LRO cilia (Yuan et al., 2015; Mizuno et al., 2020; Djenoune et al., 2023; Katoh et al., 2023). Ca^2+^ is thought to enter LRO cells through the mechanosensitive cation channel Pkd2 that localizes to immotile cilia and induce the degradation of mRNA of the Nodal antagonist Dand5. Other very recent work in the mouse LRO proposes leftward fluid flows transport extracellular vesicles (Tanaka et al., 2005) containing a Pkd2 binding partner, Pkd1l1, to the left side of the LRO to build Pkd1l1-containing fibrous structures that interact with Nodal to mediate asymmetric Ca^2+^ elevation on the left side of the LRO (Tanaka et al., 2023). Thus, while it is clear that Ca^2+^ signaling is a downstream effector of cilia-driven fluid flows in the LRO, the exact origin and regulation of asymmetric Ca^2+^ signals during LRO function have not been fully resolved.

In addition to a role(s) during LRO function, the work described here has uncovered a new role for Ca^2+^-mediated signaling much earlier in LR development as a regulator of cell proliferation during LRO formation. Our findings complement previous work that has identified multiple functions for Ca^2+^ during KV development. Inhibiting SERCA-mediated Ca^2+^ signaling using a high dose (2.5 μM) of thapsigargin for 10 min at 60% epiboly was previously found to interfere with the clustering/cohesion of DFCs during KV formation (Schneider et al., 2008). These thapsigargin treatments increased nuclear localization (activation) of the Wnt effector β-catenin. In our screen, we found that the Wnt agonist 1-Azakenpaullone (Az) that stabilizes β-catenin did not have a significant effect on DFC mitotic index (Figure 2B; Supplementary Table S2). This suggests that the reduction of DFC proliferation induced by thapsigargin is not associated with activation of the Wnt signaling pathway. An independent study found that interfering with lysophosphatidic acid signaling reduced Ca^2+^ levels and increased nuclear β-catenin in the dorsal region of the embryo during epiboly stages (Lai et al., 2012). These effects correlated with defects in DFC cohesion, malformation of KV, and a reduced number of KV cilia. In other work, treatments with a low dose (0.5 μM) of thapsigargin for 2 h during epiboly reduced mRNA expression of the T-box transcription factor *no tail* (*ntl*) and the ciliary dynein gene *dynein axonemal heavy chain 9* (or *left–right dynein related*; *lrdr*) in DFCs (Kreiling et al., 2008). These embryos also showed defective KV formation and a reduced number of KV cilia. Some of these previously reported phenotypes—including KV malformation and fewer KV cilia—are consistent with a reduced number of DFC/KV cells caused by defects in DFC proliferation. Indeed, we found small KVs and fewer KV cilia in thapsigargin treatments that suppressed the DFC mitotic index (Figure 4). Since the number of KV cilia and the number of KV cells were reduced in thapsigargin treated embryos (Figures 4C, D), we suggest that a defect in DFC proliferation provides an underlying mechanism that can explain the previously described reduced KV cilia phenotype. Downstream of KV and cilia formation, perturbations of sodium-calcium exchanger and Na/K-ATPase activities revealed that tightly regulated Ca^2+^ levels and calmodulin-dependent protein kinase II (CaMKII) activity are critical for motility of cilia in KV (Shu et al., 2007). Taken together, these studies provide evidence for multiple functions for Ca^2+^—likely via several distinct signaling pathways—during DFC/KV development.

### Ca^2+^ flux during LRO development

Here we report double transgenic *Tg*(*act2b:GCaMP6f*); *Tg*(*sox17:EGFP-caax*) zebrafish embryos that provide a new platform for imaging Ca^2+^ flux events in real-time in the developing LRO. These transgenic embryos allow visualization and quantification of Ca^2+^ transients that occur unambiguously in DFCs (Figure 6A). Transient elevation of the cytoplasmic Ca^2+^ concentration in DFCs occurred relatively frequently at the 60%–70% epiboly stages (Supplementary Table S6). Spatial mapping of cytoplasmic Ca^2+^ fluxes revealed that clusters of 2–6 neighboring DFCs experienced Ca^2+^ flux events during short imaging time windows (Supplementary Figure S3). Together, these results suggest localized regulation of cytoplasmic Ca^2+^ signaling in DFCs. However, mechanisms that may regulate such coordinated Ca^2+^ fluxes—and their physiological relevance—remain unclear. It is interesting to note that gap junctions, which can facilitate cell-to-cell Ca^2+^ signaling, have been implicated in vertebrate LR patterning (Levin and Mercola, 1998) and KV morphogenesis (Hatler et al., 2009). In future work, it would be interesting to test whether gap junctions mediate localized and/or coordinated cytoplasmic Ca^2+^ signaling in DFCs.

Live imaging of *Tg*(*act2b:GCaMP6f*); *Tg*(*sox17:EGFP-caax*) embryos also revealed nuclear Ca^2+^ signals in DFCs (Figure 7). This is the first report of nuclear Ca^2+^ transients in any vertebrate LRO. Nuclear Ca^2+^ signaling is proposed to have important functions in several cell types, which are mediated in part by regulating gene expression (Zhang et al., 2009; Bengtson and Bading, 2012). Examples include plasticity of neurons (Bading, 2000), function of cardiomyocytes (Kiessling et al., 2023), and activation of T-cells (Monaco et al., 2016). Nuclear Ca^2+^ modulates gene expression by modifying the activity of target transcription factors, such as CREB (Chawla et al., 1998), TEAD (Thompson et al., 2003), and Elk1 (Pusl et al., 2002). The nuclear envelope serves as a Ca^2+^ store and contains Ca^2+^ release channels and Ca^2+^ pumps, including SERCA, that control nuclear Ca^2+^ signaling (Mauger, 2012; Resende et al., 2013; Kiess and Kockskamper, 2019). Interestingly, nuclear Ca^2+^ signaling has been identified in some contexts as a regulator cell proliferation (Resende et al., 2013). Specifically, buffering nuclear Ca^2+^ in cultured human cell lines interfered with progression through early prophase of mitosis (Rodrigues et al., 2007). Thus, it is tempting to speculate that nuclear Ca^2+^ flux may control gene expression and/or cell cycle progression in DFCs.

Both cytoplasmic and nuclear Ca^2+^ flux events occurred in all regions of the DFC cluster, but we observed a slight bias towards the left side (Figure 6; Supplementary Figure S4; Supplementary Table S7). The functions for these Ca^2+^ events remain unknown, and it is unclear whether the leftward bias has any functional significance. To test this further, the development of new methods (possibly using optogenetic approaches) is needed to perturb Ca^2+^ flux specifically in the cytoplasm or nucleus in individual or subsets of DFCs. To assess how Ca^2+^ fluxes impact DFC morphology in unperturbed embryos, we tracked individual DFCs. Tracking DFCs for up to 20 min following a cytoplasmic Ca^2+^ transient did not reveal any gross morphological or behavioral changes. A limitation of this analysis is that we imaged cells for only 20 min, which was necessary to minimize photobleaching and phototoxicity. Tracking cells for longer periods in future work may reveal phenotypic DFC outcomes associated with cytoplasmic Ca^2+^ flux. In contrast, DFCs that experienced a nuclear Ca^2+^ flux event were found to undergo mitosis within ∼20 min (Supplementary Movie S9; Supplementary Table S9. We did not find any cell division among DFCs tracked that did not experience a nuclear Ca^2+^ flux (Supplementary Table S10). Thus, nuclear Ca^2+^ flux provides a robust proliferation marker for DFCs in living embryos. This is significant because the mechanics and regulators of DFC proliferation are not fully understood, and our current knowledge relies on static snapshots of the DFC mitotic index. For example, it is unknown whether all DFCs divide once or if there are stem-like cells that undergo multiple rounds of division to produce the final number of DFCs that give rise to KV. The identification of nuclear Ca^2+^ flux events associated with DFC mitosis opens new avenues to explore this question and ultimately define DFC mitotic dynamics in real time. We envision a new generation of transgenic tools that include expression of nuclear localized GCaMP (Forster et al., 2017) specifically in DFCs that will facilitate detection and tracking of DFC divisions in a developing embryo. Such tools could also be deployed to identify new regulators that control nuclear Ca^2+^ flux and cell division in real-time.

### Ca^2+^ signaling regulates DFC proliferation

Our work has identified SERCA-mediated Ca^2+^ signaling as a regulator of DFC proliferation during epiboly stages of development. Since thapsigargin treatments are global it remains unclear whether SERCA-mediated Ca^2+^ signaling functions cell-autonomously to control DFC proliferation. Indeed, we observed Ca^2+^ flux in dorsal margin cells neighboring DFCs and in EVL cells overlying the DFCs (Supplementary Movie S4), which could have cell non-autonomous effects on DFC proliferation. It also remains unknown whether cytoplasmic or nuclear Ca^2+^ signals (or both) are required for cell cycle progression and mitosis of DFCs. While nuclear Ca^2+^ flux was associated with DFC mitosis during live imaging, it is not clear whether nuclear Ca^2+^ flux has an active role in promoting cell division or is just a passive marker of the process. It is also possible that general Ca^2+^ homeostasis—which is disrupted by thapsigargin treatments—regulates DFC proliferation. Future work with new tools to modulate Ca^2+^ signaling in specific cell types and in specific cellular compartments will be needed to address these questions.

Mechanistically, SERCA inhibitor treatments were found to eliminate cytoplasmic and nuclear Ca^2+^ flux in DFCs (Supplementary Table S8). Previous work in several different contexts has implicated Ca^2+^ signals in regulating multiple steps of cell cycle progression. These include nuclear envelope breakdown, G1- > S transition, G2- > M transition, metaphase- > anaphase transition, and cytokinesis (Humeau et al., 2018). At the molecular level, Ca^2+^ flux can activate Calmodulin and Calmodulin dependent protein kinases (CaMK), which can result in the modification of cell cycle proteins, including Retinoblastoma protein (Rb) (Morris et al., 1998; Rodriguez-Mora et al., 2005), cyclin-dependent kinases (Cdks) (Morris et al., 1998; Kahl and Means, 2004), and the Cdc25C phosphatase (Patel et al., 1999). In addition, Calmodulin can activate the protein phosphatase Calcineurin that controls cell cycle gene expression and cyclin protein levels (Masaki and Shimada, 2022). While it is clear that Ca^2+^ signaling has important functions during the cell cycle, results from different models and contexts indicate Ca^2+^ signals are used differently in different types of cells to regulate cell cycle progression (Machaca, 2011). Thus, we investigated the cellular mechanism by which SERCA inhibitor treatments reduced the DFC mitotic index. Blocking SERCA activity in human cell lines was previously found to impede the transition between G1 and S phases (Simon and Moran, 2001). However, in DFCs, we find that inhibiting SERCA impedes progression of DFCs through the S/G2 phases of the cell cycle (Figure 5). This is important because it identifies a specific defect in the cell cycle that sets up future work to characterize molecular details for how Ca^2+^ signaling functions to regulate DFC proliferation. Interestingly, inhibiting CaMKII activity results in cells accumulating in the G2 or M phases (Skelding et al., 2011), and altering CaMKII function in zebrafish results in LR asymmetry defects (Francescatto et al., 2010). CaMKII activity is therefore an attractive candidate as a downstream effector of SERCA-mediated Ca^2+^ flux during DFC proliferation.

In conclusion, our results identify the SERCA-mediated Ca^2+^ signaling as a new regulator of DFC mitosis. SERCA inhibitor treatments reduced the number of ciliated KV cells and disrupted embryo laterality. At the molecular level we found that inhibiting SERCA reduced both cytoplasmic and nuclear Ca^2+^ flux, and at the cellular level Ca^2+^ signaling controls progression of DFCs through the S/G2 phases of the cell cycle. These results uncover a previously unrecognized role for SERCA-mediated Ca^2+^ signaling in regulating the proliferation of precursor cells that give rise to the ciliated LRO. This finding adds to the growing number of functions for Ca^2+^ signaling during LR axis determination.

## Materials and methods

### Zebrafish husbandry

Adult zebrafish (*Danio rerio*) were maintained in an aquarium rack system (Aquatic Habitats, LLC) on a 14 h light/10 h dark cycle. The wild-type TAB strain was acquired from Zebrafish International Resource Center (ZIRC). Transgenic strains used in this study include *Tg*(*sox17:EGFP-CAAX,myl7:EGFP*)^*sny101*^ (Dasgupta et al., 2018), *Tg*(*-3.5ubb:Cerulean-gmnn-2A-mCherry-cdt1*)^*w141*^ [referred to here as *Tg*(*Dual-FUCCI*)] (Bouldin et al., 2014) kindly provided by Donald Kane, and *Tg*(*actb2:GCaMP6*)^*sny210*^ (generated in this study). Zebrafish embryos were collected from natural matings, staged according to (Kimmel et al., 1995). A Ziess Discovery V12 Stereo microscope equipped with X-cite Fluorescence Illumination and a Zeiss Axiocam MRc digital camera was used to visualize living embryos and assess developmental stage, gross morphology, or heart laterality. All experiments were approved by State University of New York Upstate Medical University’s Institutional Animal Care and Use Committee.

### Pharmacological screen

Embryos were treated with selected pharmacological compounds in 24-well plates (Corning). ∼15 embryos in chorions were treated per well starting at the 60% epiboly stage (6.5 hpf) for 60 min. The working concentration and manufacturer for each compound is reported in Supplementary Table S1. Since the stock solution of each compound was made using dimethyl sulfoxide (DMSO), negative control embryos were treated with 1% DMSO (an excess of DMSO relative to pharmacological compound dilutions) diluted in embryo medium (reverse osmosis purified water with 0.03% marine salt from Instant Ocean and 0.001% methylene blue from Sigma). Following the 60 min treatment, the compounds were removed and the embryos were washed 3X with embryo medium. To assess DFC proliferation rates, treated embryos were fixed immediately at 7.5 hpf (70% epiboly stage) for fluorescent immunostaining using GFP and pHH3 antibodies (see below). In some cases, treated embryos were imaged to quantify Ca^2+^ flux events in DFCs (see below) or visualized the next day to assess heart laterality defects.

### Immunostaining

Fluorescent immunostaining was performed as previously described (Santra and Amack, 2021). Embryos were fixed in 4% paraformaldehyde +1% tween-20 in 1X phosphate-buffered saline (PBS) overnight at 4°C, and then dechorionated and permeabilized in 100% acetone at −20°C for 8 min. Next, the embryos were incubated in blocking solution (10% bovine serum albumin in 1X PBS) for 1 h at room temperature. Primary antibodies were diluted in blocking solution and incubated with embryos at 4°C overnight. Primary antibodies used in this study were anti-EGFP (GeneTex, GTX13970) diluted to 1:400, anti-phosphorylated Histone H3 (pHH3; Cell Signaling Technology, 9701S) diluted to 1:400, anti-mCherry (Abcam, ab183628) diluted to 1:200, and anti-acetylated tubulin (Sigma, T6793) diluted to 1:400. After the primary antibodies were removed, the embryos were washed in 1X PBS +1% Tween-20 (8 × 15 min washes) at room temperature and then incubated in blocking solution for 1 h at room temperature. Fluorescent secondary antibodies, Alexa Fluor 488 or 568 (Invitrogen) diluted 1:200 in blocking solution, were incubated with embryos at 4°C overnight. DAPI (Sigma) was also included at a dilution of 1:500. Embryos were imaged using either a Zeiss Axio Imager M1 compound microscope using a ×63 objective, or a Perkin-Elmer Ultra View Vox spinning disk confocal microscope using a ×40 objective, or a Nikon spinning disk confocal system (Yokogawa CSU-X1) using a ×40 objective.

### Quantification of DFCs and KV cells

Immunostaining images were blinded and quantitatively analyzed using FIJI (NIH) software. To quantify DFC proliferation, the number of pHH3 positive DFCs and the total number of DFCs were manually counted in *Tg*(*sox17:EGFP-caax*) embryos as previously described (Gokey et al., 2015). The number of pHH3 positive DFCs was divided by the total number of DFCs to calculate the DFC mitotic index. To quantify the mitotic index of cells neighboring DFCs, a boundary was drawn around the DFC cluster and then pasted onto the field cells above the DFCs in the embryo (these are dorsal margin cells). The mitotic index of the dorsal margin cells within the boundary was calculated. To determine the area of KV, maximum projections of confocal images of EGFP immunostained *Tg*(*sox17:EGFP-caax*) embryos were used to manually trace the perimeter of KV in FIJI and measure the area. To quantify the number of cilia or cells in KV, acetylated tubulin positive cilia or DAPI stained nuclei in EGFP positive KV cells were manually counted in *Tg*(*sox17:EGFP-caax*) embryos using confocal Z-series through KV (Tay et al., 2013). For these quantitative analyses, drug treated embryos pooled from at least two independent experiments were compared to control DMSO treated embryos from the same experiments using an unpaired *t*-test with Welch’s correction.

### RNA *in situ* hybridizations

For RNA *in situ* hybridizations, we followed a previously described protocol (Gao et al., 2010). An RNA probe complimentary to the *southpaw* (*spaw*) coding sequence was labeled with digoxygenin using a Roche DIG RNA labeling kit. Embryos were fixed using 4% paraformaldehyde. The fixed embryos were then incubated in a prehybridization solution, containing formamide, SSC (saline-sodium citrate), and blocking reagents. The probe was then added to the embryos and allowed to hybridize to the target mRNA at 60°C overnight. After hybridization, the samples are washed to remove the unbound probe using a series of stringent washes in SSC buffer with or without formamide. A Roche anti-digoxigenin antibody conjugated to alkaline phosphatase (Sigma) was used at a 1:1000 dilution for detection of digoxigenin and incubated at 4°C overnight. After a series of washes, alkaline phosphatase activity was detected via a colorimetric assay using NBT and BCIP (Roche). Images were acquired using a Ziess Discovery V12 Stereo microscope equipped with a Zeiss Axiocam MRc digital camera.

### Cell cycle analysis

To assess cell cycle status in DFCs, *Tg*(*Dual-FUCCI*); *Tg*(*sox17:EGFP-caax*) embryos were fixed at specific developmental stages and fluorescently stained (see Immunostaining protocol details above) using anti-mCherry antibodies that recognize mCherry-Cdt1 and anti-EGFP antibodies that detect cerulean-Geminin. Images of the embryos were captured using a Perkin-Elmer Ultra View Vox spinning disk confocal microscope using a ×40 objective. FIJI (NIH) software was used to manually count the total number of DAPI stained nuclei in EGFP positive DFCs in a confocal Z-series through the DFC cluster, as well as the number of DFC nuclei with mCherry > EGFP staining intensity (defined as cells in G1) and EGFP > mCherry staining (defined as cells in S/G2/M). Cells with condensed DNA were included in the S/G2/M group. Treatments were compared using an unpaired *t*-test with Welch’s correction.

### Generation of transgenic *Tg*(*actb2:GCaMP6*) zebrafish

To create a transgenic Ca^2+^ reporter zebrafish strain, we utilized Tol2 transgenesis technology and the GCaMP6f calcium sensor. Gateway cloning was performed as described in (Kwan et al., 2007) to assemble the Tol2 act2b-GCaMP6-pA transgene construct. A construct containing GCaMP6f (Chen et al., 2013) cDNA was a kind gift from David Raible, and the beta-actin 2 (*actb2*) promoter and polyadenylation (pA) sequences were obtained from the Tol2kit (Kwan et al., 2007). The Tol2 transgene (plasmid DNA) was injected into 1-cell wild-type TAB embryos with mRNA encoding Tol2 transposase enzyme. These embryos were screened for GCaMP6f positive founders as identified by GFP signal, and positive embryos were raised to adulthood. Founders were then out-crossed with wild-type TAB and the resulting embryos were screened for GFP positive signal to identify F1 fish. The brightest F1 embryos were raised to establish the *Tg*(*actb2:GCaMP6*)^*sny210*^ transgenic line.

### Visualization and quantification of Ca^2+^ flux events in DFCs

Live double transgenic *Tg*(*sox17:EGFP-CAAX,myl7:EGFP*)*;Tg*(*actb2:GCaMP6*) embryos in the chorion were immobilized in 1% agarose and imaged using a Perkin-Elmer Ultra View Vox spinning disk confocal microscope with a ×40 objective. A Z-series of images through the DFCs were captured every 15 s for 10–40 min. To determine the duration of cytoplasmic Ca^2+^ flux events, fluorescence intensity profiles were plotted for individual cells using FIJI (NIH) software to identify the start of a Ca^2+^ flux event (defined as the timepoint when the cytoplasmic GCaMP6f fluorescence intensity increased above baseline levels) and end of the event (defined as the timepoint when fluorescence intensity returned to baseline). To estimate the number of Ca^2+^ flux events that occurred in the DFC cluster over a defined time period, transient increases in GCaMP6 fluorescence intensity occurring in DFCs were manually counted for each Z-stack at every time point using Volocity (Perkin Elmer) or FIJI (NIH) software. To approximate the relative position of Ca^2+^ flux events in DFCs, the location of individual cells undergoing either cytoplasmic or nuclear Ca^2+^ flux were superimposed onto a maximum projection of the DFC cluster from each embryo analyzed. The DFC cluster was divided into quadrants based on the overall length and height of the cluster. Results were pooled from 10 embryos to determine the percentage of events in each quadrant.

### Statistical analysis

For all data sets, an unpaired two-tailed *t*-test with Welch’s correction was used to calculate *p* values. A *p*-value less than 0.01 was considered a significant difference.

## Data Availability

The raw data supporting the conclusion of this article will be made available by the authors, without undue reservation.

## Supplementary material

SUPPLEMENTARY FIGURE S1 SERCA inhibitor treatments do not alter the mitotic index of non-DFCs at the dorsal margin. **(A)** Representative merged images of pHH3 staining in *Tg(sox17:EGFP-caax)* embryos treated with 1% DMSO (control) or 1 μM Thaps at 60% epiboly for 60 min. **(B)** Bar graphs indicate average mitotic index of neighboring non-DFCs in DMSO (control) and 1 μM Thaps treated embryos and error bars represent one standard deviation. Each circle on the graph represents results from an individual embryo. An unpaired two-tailed *t*-test with Welch’s correction was used for statistical analysis. ns = not significant (*p* = 0.4446).

SUPPLEMENTARY FIGURE S2 SERCA inhibitor treatments during epiboly alters heart looping. **(A–B)** Embryos at 2 days post-fertilization that were treated with 1% DMSO (control) **(A)** or 1 μM thapsigargin **(B)** at 60% epiboly for 60 min. **(C–E)** Representative images of normal rightward looping of the heart (arrow) in a control embryo **(C)**, and midline **(D)** or reversed **(E)** looping in thapsigargin treated embryos. The heart was labeled by EGFP expression in cardiomyocytes. V = ventricle; A = atrium. **(F)** Rightward heart looping was observed in most control embryos, whereas the heart often remained along the midline or looped to the left in thapsigargin treated embryos. n = number of embryos analyzed.

SUPPLEMENTARY FIGURE S3 Spatial location of DFC cytoplasmic Ca^2+^ flux events in individual embryos. Solid colored lines indicate the boundary of the DFC cluster in an individual embryo as determined by confocal images (see Figure 6B). The DFC cluster was divided into quadrants based on the overall length and height of the cluster. Colored ovals indicate the location of cytoplasmic Ca^2+^ flux events. Dashed lines group clusters of DFC cytoplasmic Ca^2+^ flux events. The DFC cluster for all 5 embryos were overlayed to create Figure 6C. L = left, R = right, A = anterior, P = posterior.

SUPPLEMENTARY FIGURE S4 Spatial location of DFC nuclear Ca^2+^ fluxes in individual embryos. **(A)** Solid colored lines indicate the boundary of the DFC cluster in an individual embryo as determined by confocal images (see Figure 7B). The DFC cluster was divided into quadrants based on the overall length and height of the cluster. Colored ovals indicate the location of nuclear Ca^2+^ flux events. The DFC cluster for all 5 embryos were overlayed to create Figure 7C. **(B–D)** Location of DFC nuclear Ca^2+^ flux events in DFC quadrants **(B)**, along the LR axis **(C)**, and along the AP axis **(D)**. L = left, R = right, A = anterior, P = posterior.

SUPPLEMENTARY MOVIE S1 Visualization of Ca^2+^ dynamics in a *Tg(act2b:GCaMP6f)* embryonic heart. Confocal optical section through the beating embryonic heart detects transient increases in Ca^2+^ concentration (GCaMP6f fluorescence intensity) in cardiomyocytes visualized using the 16-colors lookup table (FIJI software).

SUPPLEMENTARY MOVIE S2 Visualization of Ca^2+^ dynamics in a *Tg(act2b:GCaMP6f)* embryonic olfactory pit. Confocal imaging of the embryonic olfactory pit detects transient increases in Ca^2+^ concentration (GCaMP6f fluorescence intensity) visualized using the 16-colors lookup table (FIJI software).

SUPPLEMENTARY MOVIE S3 Visualization of Ca^2+^ dynamics in a *Tg(act2b:GCaMP6f)* embryonic neuromast. Confocal imaging of an embryonic neuromast detects transient increases in Ca^2+^ concentration (GCaMP6f fluorescence intensity) visualized using the 16-colors lookup table (FIJI software).

SUPPLEMENTARY MOVIE S4 Visualization of Ca^2+^ dynamics in DFCs. Time-lapse confocal imaging of Ca^2+^ flux events in wild-type *Tg(act2b:GCaMP6f)*; *Tg(sox17:EGFP-caax)* embryos starting at the 60% epiboly stage. DFC cell membranes are labeled by *Tg(sox17:EGFPcaax)* expression, and changes in Ca^2+^ concentration (GCaMP6f fluorescence intensity) are visualized using the cyan hot lookup table (FIJI software). This is a maximum projection of the entire DFC cluster. Timestamp = hours:minutes:seconds:milliseconds.

SUPPLEMENTARY MOVIE S5 Visualization of Ca^2+^ dynamics in DMSO control treated DFCs. Timelapse confocal imaging of Ca^2+^ flux events in *Tg(act2b:GCaMP6f)*; *Tg(sox17:EGFPcaax)* embryos treated with 1% DMSO at the 60% epiboly stage for 60 min. DFCs were imaged at at the 70% epiboly stage. DFC cell membranes are labeled by *Tg(sox17:EGFP-caax)* expression, and changes in Ca^2+^ concentration (GCaMP6f fluorescence intensity) are visualized using the cyan hot lookup table (FIJI software). This is a maximum projection of the entire DFC cluster. Timestamp = hours:minutes:seconds:milliseconds.

SUPPLEMENTARY MOVIE S6 Visualization of Ca^2+^ dynamics in thapsigargin treated DFCs. Time-lapse confocal imaging of Ca^2+^ flux events in *Tg(act2b:GCaMP6f)*; *Tg(sox17:EGFP-caax)* embryos treated with 1 μM thapsigargin at the 60% epiboly stage for 60 min. DFCs were imaged at at the 70% epiboly stage. DFC cell membranes are labeled by *Tg(sox17:EGFP-caax)* expression, and changes in Ca^2+^ concentration (GCaMP6f fluorescence intensity) are visualized using the cyan hot lookup table (FIJI software). This is a maximum projection of the entire DFC cluster. Timestamp = hours:minutes:seconds:milliseconds.

SUPPLEMENTARY MOVIE S7 Visualization of Ca^2+^ dynamics in cyclopiazonic acid treated DFCs. Time-lapse confocal imaging of Ca^2+^ flux events in *Tg(act2b:GCaMP6f)*; *Tg(sox17:EGFP-caax)* embryos treated with 100 μM cyclopiazonic acid at the 60% epiboly stage for 60 min. DFCs were imaged at at the 70% epiboly stage. DFC cell membranes are labeled by *Tg(sox17:EGFP-caax)* expression, and changes in Ca^2+^ concentration (GCaMP6f fluorescence intensity) are visualized using the cyan hot lookup table (FIJI software). This is a maximum projection of the entire DFC cluster. Timestamp = hours:minutes:seconds:milliseconds.

SUPPLEMENTARY MOVIE S8 DFC behavior following cytoplasmic Ca^2+^ fluxes. The behavior of a DFC (arrow) in a *Tg(act2b:GCaMP6f)*; *Tg(sox17:EGFP-caax)* embryo that undergoes multiple cytoplasmic Ca^2+^ flux events (asterisks) was tracked for 20 min. These cytoplasmic Ca^2+^ flux events did not result in gross changes in DFC morphology, behavior, or position in this timeframe. This is a single focal plane within the DFC cluster. Timestamp = minutes:seconds.

SUPPLEMENTARY MOVIE S9 DFC mitosis following nuclear Ca^2+^ flux. The behavior of a DFC (arrow) in a *Tg(act2b:GCaMP6f)*; *Tg(sox17:EGFP-caax)* embryo that undergoes a nuclear Ca^2+^ flux event (asterisk) was tracked for 20 min. The DFC rounded up and divided into two daughter cells. This is a single focal plane within the DFC cluster. Timestamp = minutes:seconds.

## References

[B1] Afzelius B. A. (1976). A human syndrome caused by immotile cilia. Science 193 (4250), 317–319. 10.1126/science.1084576 1084576

[B3] Amack J. D. (2022). Structures and functions of cilia during vertebrate embryo development. Mol. Reprod. Dev. 89, 579–596. 10.1002/mrd.23650 36367893 PMC9805515

[B4] Amack J. D. Wang X. Yost H. J. (2007). Two T-box genes play independent and cooperative roles to regulate morphogenesis of ciliated Kupffer's vesicle in zebrafish. Dev. Biol. 310 (2), 196–210. 10.1016/j.ydbio.2007.05.039 17765888

[B5] Amack J. D. Yost H. J. (2004). The T box transcription factor no tail in ciliated cells controls zebrafish left-right asymmetry. Curr. Biol. 14 (8), 685–690. 10.1016/j.cub.2004.04.002 15084283

[B6] Arrington C. B. Peterson A. G. Yost H. J. (2013). Sdc2 and Tbx16 regulate Fgf2-dependent epithelial cell morphogenesis in the ciliated organ of asymmetry. Development 140 (19), 4102–4109. 10.1242/dev.096933 24046323 PMC3775420

[B7] Aylsworth A. S. (2001). Clinical aspects of defects in the determination of laterality. Am. J. Med. Genet. 101 (4), 345–355. 10.1002/ajmg.1219 11471158

[B8] Bading H. (2000). Transcription-dependent neuronal plasticity: the nuclear calcium hypothesis. Eur. J. Biochem. 267 (17), 5280–5283. 10.1046/j.1432-1327.2000.01565.x 10951185

[B9] Bengtson C. P. Bading H. (2012). Nuclear calcium signaling. Adv. Exp. Med. Biol. 970, 377–405. 10.1007/978-3-7091-0932-8_17 22351065

[B10] Blum M. Andre P. Muders K. Schweickert A. Fischer A. Bitzer E. (2007). Ciliation and gene expression distinguish between node and posterior notochord in the mammalian embryo. Differentiation 75 (2), 133–146. 10.1111/j.1432-0436.2006.00124.x 17316383

[B11] Blum M. Vick P. (2015). Left-right asymmetry: cilia and calcium revisited. Curr. Biol. 25 (5), R205–R207. 10.1016/j.cub.2015.01.031 25734272

[B12] Blum M. Weber T. Beyer T. Vick P. (2009). Evolution of leftward flow. Semin. Cell. Dev. Biol. 20 (4), 464–471. 10.1016/j.semcdb.2008.11.005 19056505

[B13] Bootman M. D. Bultynck G. (2020). Fundamentals of cellular calcium signaling: a primer. Cold Spring Harb. Perspect. Biol. 12 (1), a038802. 10.1101/cshperspect.a038802 31427372 PMC6942118

[B14] Bouldin C. M. Snelson C. D. Farr G. H. Kimelman D. (2014). Restricted expression of cdc25a in the tailbud is essential for formation of the zebrafish posterior body. Genes. Dev. 28 (4), 384–395. 10.1101/gad.233577.113 24478331 PMC3937516

[B15] Buca D. I. P. Khalil A. Rizzo G. Familiari A. Di Giovanni S. Liberati M. (2018). Outcome of prenatally diagnosed fetal heterotaxy: systematic review and meta-analysis. Ultrasound Obstet. Gynecol. 51 (3), 323–330. 10.1002/uog.17546 28603940

[B19] Chawla S. Hardingham G. E. Quinn D. R. Bading H. (1998). CBP: a signal-regulated transcriptional coactivator controlled by nuclear calcium and CaM kinase IV. Science 281 (5382), 1505–1509. 10.1126/science.281.5382.1505 9727976

[B20] Chen J. Xia L. Bruchas M. R. Solnica-Krezel L. (2017). Imaging early embryonic calcium activity with GCaMP6s transgenic zebrafish. Dev. Biol. 430 (2), 385–396. 10.1016/j.ydbio.2017.03.010 28322738 PMC5835148

[B21] Chen J. N. van Eeden F. J. Warren K. S. Chin A. Nüsslein-Volhard C. Haffter P. (1997). Left-right pattern of cardiac BMP4 may drive asymmetry of the heart in zebrafish. Development 124 (21), 4373–4382. 10.1242/dev.124.21.4373 9334285

[B22] Chen T. W. Wardill T. J. Sun Y. Pulver S. R. Renninger S. L. Baohan A. (2013). Ultrasensitive fluorescent proteins for imaging neuronal activity. Nature 499 (7458), 295–300. 10.1038/nature12354 23868258 PMC3777791

[B24] Clapham D. E. (2007). Calcium signaling. Cell. 131 (6), 1047–1058. 10.1016/j.cell.2007.11.028 18083096

[B25] Collado-Hilly M. Shirvani H. Jaillard D. Mauger J. P. (2010). Differential redistribution of Ca2+-handling proteins during polarisation of MDCK cells: effects on Ca2+ signalling. Cell. Calcium 48 (4), 215–224. 10.1016/j.ceca.2010.09.003 20932574

[B26] Cooper M. S. D’Amico L. A. (1996). A cluster of noninvoluting endocytic cells at the margin of the zebrafish blastoderm marks the site of embryonic shield formation. Dev. Biol. 180 (1), 184–198. 10.1006/dbio.1996.0294 8948584

[B27] Dasgupta A. Amack J. D. (2016). Cilia in vertebrate left-right patterning. Philos. Trans. R. Soc. Lond B Biol. Sci. 371 (1710), 20150410. 10.1098/rstb.2015.0410 27821522 PMC5104509

[B28] Dasgupta A. Merkel M. Clark M. J. (2018). Cell volume changes contribute to epithelial morphogenesis in zebrafish Kupffer's vesicle. Elife 7, e30963. 10.7554/eLife.30963 29376824 PMC5800858

[B29] Desgrange A. Le Garrec J. F. Meilhac S. M. (2018). Left-right asymmetry in heart development and disease: forming the right loop. Development 145 (22), dev162776. 10.1242/dev.162776 30467108

[B30] Djenoune L. Mahamdeh M. Truong T. V. Nguyen C. T. Fraser S. E. Brueckner M. (2023). Cilia function as calcium-mediated mechanosensors that instruct left-right asymmetry. Science 379 (6627), 71–78. 10.1126/science.abq7317 36603098 PMC9939240

[B31] Essner J. J. Amack J. D. Nyholm M. K. Harris E. B. Yost H. J. (2005). Kupffer's vesicle is a ciliated organ of asymmetry in the zebrafish embryo that initiates left-right development of the brain, heart and gut. Development 132 (6), 1247–1260. 10.1242/dev.01663 15716348

[B32] Essner J. J. Vogan K. J. Wagner M. K. Tabin C. J. Yost H. J. Brueckner M. (2002). Conserved function for embryonic nodal cilia. Nature 418 (6893), 37–38. 10.1038/418037a 12097899

[B33] Forrest K. Barricella A. C. Pohar S. A. Hinman A. M. Amack J. D. (2022). Understanding laterality disorders and the left-right organizer: insights from zebrafish. Front. Cell. Dev. Biol. 10, 1035513. 10.3389/fcell.2022.1035513 36619867 PMC9816872

[B34] Forster D. Dal Maschio M. Laurell E. Baier H. (2017). An optogenetic toolbox for unbiased discovery of functionally connected cells in neural circuits. Nat. Commun. 8 (1), 116. 10.1038/s41467-017-00160-z 28740141 PMC5524645

[B35] Francescatto L. Rothschild S. C. Myers A. L. Tombes R. M. (2010). The activation of membrane targeted CaMK-II in the zebrafish Kupffer's vesicle is required for left-right asymmetry. Development 137 (16), 2753–2762. 10.1242/dev.049627 20630945

[B36] Gao C. Wang G. Amack J. D. Mitchell D. R. (2010). Oda16/Wdr69 is essential for axonemal dynein assembly and ciliary motility during zebrafish embryogenesis. Dev. Dyn. 239 (8), 2190–2197. 10.1002/dvdy.22355 20568242 PMC3077675

[B37] Garic-Stankovic A. Hernandez M. Flentke G. R. Zile M. H. Smith S. M. (2008). A ryanodine receptor-dependent Ca(i)(2+) asymmetry at Hensen's node mediates avian lateral identity. Development 135 (19), 3271–3280. 10.1242/dev.018861 18755776 PMC2999519

[B39] Gokey J. J. Dasgupta A. Amack J. D. (2015). The V-ATPase accessory protein Atp6ap1b mediates dorsal forerunner cell proliferation and left-right asymmetry in zebrafish. Dev. Biol. 407 (1), 115–130. 10.1016/j.ydbio.2015.08.002 26254189 PMC4641761

[B40] Hamada H. (2020). Molecular and cellular basis of left-right asymmetry in vertebrates. Proc. Jpn. Acad. Ser. B Phys. Biol. Sci. 96 (7), 273–296. 10.2183/pjab.96.021 PMC7443379 32788551

[B41] Hans F. Dimitrov S. (2001). Histone H3 phosphorylation and cell division. Oncogene 20 (24), 3021–3027. 10.1038/sj.onc.1204326 11420717

[B42] Hatayama M. Mikoshiba K. Aruga J. (2011). IP3 signaling is required for cilia formation and left-right body axis determination in Xenopus embryos. Biochem. Biophys. Res. Commun. 410 (3), 520–524. 10.1016/j.bbrc.2011.06.014 21683063

[B43] Hatler J. M. Essner J. J. Johnson R. G. (2009). A gap junction connexin is required in the vertebrate left-right organizer. Dev. Biol. 336 (2), 183–191. 10.1016/j.ydbio.2009.09.035 19799895

[B46] Humeau J. Bravo-San Pedro J. M. Vitale I. Nuñez L. Villalobos C. Kroemer G. (2018). Calcium signaling and cell cycle: progression or death. Cell. Calcium 70, 3–15. 10.1016/j.ceca.2017.07.006 28801101

[B48] Jurynec M. J. Xia R. Mackrill J. J. Gunther D. Crawford T. Flanigan K. M. (2008). Selenoprotein N is required for ryanodine receptor calcium release channel activity in human and zebrafish muscle. Proc. Natl. Acad. Sci. U. S. A. 105 (34), 12485–12490. 10.1073/pnas.0806015105 18713863 PMC2527938

[B49] Kahl C. R. Means A. R. (2004). Regulation of cyclin D1/Cdk4 complexes by calcium/calmodulin-dependent protein kinase I. J. Biol. Chem. 279 (15), 15411–15419. 10.1074/jbc.M312543200 14754892

[B50] Katoh T. A. Omori T. Mizuno K. Sai X. Minegishi K. Ikawa Y. (2023). Immotile cilia mechanically sense the direction of fluid flow for left-right determination. Science 379 (6627), 66–71. 10.1126/science.abq8148 36603091

[B51] Kiess T. O. Kockskamper J. (2019). SERCA activity controls the systolic calcium increase in the nucleus of cardiac myocytes. Front. Physiol. 10, 56. 10.3389/fphys.2019.00056 30787882 PMC6373492

[B52] Kiessling M. Djalinac N. Voglhuber J. Ljubojevic-Holzer S. (2023). Nuclear calcium in cardiac (Patho)Physiology: small compartment, big impact. Biomedicines 11 (3), 960. 10.3390/biomedicines11030960 36979939 PMC10046765

[B53] Kim S. Zaghloul N. A. Bubenshchikova E. Oh E. C. Rankin S. Katsanis N. (2011). Nde1-mediated inhibition of ciliogenesis affects cell cycle re-entry. Nat. Cell. Biol. 13 (4), 351–360. 10.1038/ncb2183 21394081 PMC3077088

[B54] Kimmel C. B. Ballard W. W. Kimmel S. R. Ullmann B. Schilling T. F. (1995). Stages of embryonic development of the zebrafish. Dev. Dyn. 203 (3), 253–310. 10.1002/aja.1002030302 8589427

[B55] Kramer-Zucker A. G. Olale F. Haycraft C. J. Yoder B. K. Schier A. F. Drummond I. A. (2005). Cilia-driven fluid flow in the zebrafish pronephros, brain and Kupffer's vesicle is required for normal organogenesis. Development 132 (8), 1907–1921. 10.1242/dev.01772 15790966

[B56] Kreiling J. A. Balantac Z. L. Crawford A. R. Ren Y. Toure J. Zchut S. (2008). Suppression of the endoplasmic reticulum calcium pump during zebrafish gastrulation affects left-right asymmetry of the heart and brain. Mech. Dev. 125 (5-6), 396–410. 10.1016/j.mod.2008.02.004 18359204

[B57] Kwan K. M. Fujimoto E. Grabher C. Mangum B. D. Hardy M. E. Campbell D. S. (2007). The Tol2kit: a multisite gateway-based construction kit for Tol2 transposon transgenesis constructs. Dev. Dyn. 236 (11), 3088–3099. 10.1002/dvdy.21343 17937395

[B58] Lai S. L. Yao W. L. Tsao K. C. Houben A. J. S. Albers H. M. H. G. Ovaa H. (2012). Autotaxin/Lpar3 signaling regulates Kupffer's vesicle formation and left-right asymmetry in zebrafish. Development 139 (23), 4439–4448. 10.1242/dev.081745 23095890

[B59] Langenbacher A. Chen J. N. (2008). Calcium signaling: a common thread in vertebrate left-right axis development. Dev. Dyn. 237 (12), 3491–3496. 10.1002/dvdy.21704 18792114 PMC2836160

[B60] Lanini L. Bachs O. Carafoli E. (1992). The calcium pump of the liver nuclear membrane is identical to that of endoplasmic reticulum. J. Biol. Chem. 267 (16), 11548–11552. 10.1016/s0021-9258(19)49945-6 1317870

[B61] Levin M. Mercola M. (1998). Gap junctions are involved in the early generation of left-right asymmetry. Dev. Biol. 203 (1), 90–105. 10.1006/dbio.1998.9024 9806775

[B62] Little R. B. Norris D. P. (2021). Right, left and cilia: how asymmetry is established. Semin. Cell. Dev. Biol. 110, 11–18. 10.1016/j.semcdb.2020.06.003 32571625

[B63] Liu J. Zhu C. Ning G. Yang L. Cao Y. Huang S. (2019). Chemokine signaling links cell-cycle progression and cilia formation for left-right symmetry breaking. PLoS Biol. 17 (8), e3000203. 10.1371/journal.pbio.3000203 31430272 PMC6716676

[B64] Long S. Ahmad N. Rebagliati M. (2003). The zebrafish nodal-related gene southpaw is required for visceral and diencephalic left-right asymmetry. Development 130 (11), 2303–2316. 10.1242/dev.00436 12702646

[B66] Lytton J. Westlin M. Hanley M. R. (1991). Thapsigargin inhibits the sarcoplasmic or endoplasmic reticulum Ca-ATPase family of calcium pumps. J. Biol. Chem. 266 (26), 17067–17071. 10.1016/s0021-9258(19)47340-7 1832668

[B67] Machaca K. (2011). Ca(2+) signaling, genes and the cell cycle. Cell. Calcium 49 (5), 323–330. 10.1016/j.ceca.2011.05.004 21809493

[B68] Masaki T. Shimada M. (2022). Decoding the phosphatase code: regulation of cell proliferation by Calcineurin. Int. J. Mol. Sci. 23 (3), 1122. 10.3390/ijms23031122 35163061 PMC8835043

[B70] Mauger J. P. (2012). Role of the nuclear envelope in calcium signalling. Biol. Cell. 104 (2), 70–83. 10.1111/boc.201100103 22188206

[B71] McGrath J. Somlo S. Makova S. Tian X. Brueckner M. (2003). Two populations of node monocilia initiate left-right asymmetry in the mouse. Cell. 114 (1), 61–73. 10.1016/s0092-8674(03)00511-7 12859898

[B72] Melby A. E. Warga R. M. Kimmel C. B. (1996). Specification of cell fates at the dorsal margin of the zebrafish gastrula. Development 122 (7), 2225–2237. 10.1242/dev.122.7.2225 8681803

[B74] Mizuno K. Shiozawa K. Katoh T. A. Minegishi K. Ide T. Ikawa Y. (2020). Role of Ca(2+) transients at the node of the mouse embryo in breaking of left-right symmetry. Sci. Adv. 6 (30), eaba1195. 10.1126/sciadv.aba1195 32743070 PMC7375832

[B75] Monaco S. Jahraus B. Samstag Y. Bading H. (2016). Nuclear calcium is required for human T cell activation. J. Cell. Biol. 215 (2), 231–243. 10.1083/jcb.201602001 27810914 PMC5084645

[B76] Morris T. A. DeLorenzo R. J. Tombes R. M. (1998). CaMK-II inhibition reduces cyclin D1 levels and enhances the association of p27kip1 with Cdk2 to cause G1 arrest in NIH 3T3 cells. Exp. Cell. Res. 240 (2), 218–227. 10.1006/excr.1997.3925 9596994

[B78] Nonaka S. Tanaka Y. Okada Y. Takeda S. Harada A. Kanai Y. (1998). Randomization of left-right asymmetry due to loss of nodal cilia generating leftward flow of extraembryonic fluid in mice lacking KIF3B motor protein. Cell. 95 (6), 829–837. 10.1016/s0092-8674(00)81705-5 9865700

[B79] Norris D. P. (2012). Cilia, calcium and the basis of left-right asymmetry. BMC Biol. 10, 102. 10.1186/1741-7007-10-102 23256866 PMC3527145

[B80] Okada Y. Takeda S. Tanaka Y. Belmonte J. C. I. Hirokawa N. (2005). Mechanism of nodal flow: a conserved symmetry breaking event in left-right axis determination. Cell. 121 (4), 633–644. 10.1016/j.cell.2005.04.008 15907475

[B81] Oteiza P. Köppen M. Concha M. L. Heisenberg C. P. (2008). Origin and shaping of the laterality organ in zebrafish. Development 135 (16), 2807–2813. 10.1242/dev.022228 18635607

[B82] Patel R. Holt M. Philipova R. Moss S. Schulman H. Hidaka H. (1999). Calcium/calmodulin-dependent phosphorylation and activation of human Cdc25-C at the G2/M phase transition in HeLa cells. J. Biol. Chem. 274 (12), 7958–7968. 10.1074/jbc.274.12.7958 10075693

[B83] Primeau J. O. Armanious G. P. Fisher M. E. Young H. S. (2018). The SarcoEndoplasmic reticulum calcium ATPase. Subcell. Biochem. 87, 229–258. 10.1007/978-981-10-7757-9_8 29464562

[B84] Pusl T. Wu J. J. Zimmerman T. L. Zhang L. Ehrlich B. E. Berchtold M. W. (2002). Epidermal growth factor-mediated activation of the ETS domain transcription factor Elk-1 requires nuclear calcium. J. Biol. Chem. 277 (30), 27517–27527. 10.1074/jbc.M203002200 11971908

[B85] Rathbun L. I. Colicino E. G. Manikas J. O'Connell J. Krishnan N. Reilly N. S. (2020). Cytokinetic bridge triggers *de novo* lumen formation *in vivo* . Nat. Commun. 11 (1), 1269. 10.1038/s41467-020-15002-8 32152267 PMC7062744

[B86] Raya A. Kawakami Y. Rodríguez-Esteban C. Ibañes M. Rasskin-Gutman D. Rodríguez-León J. (2004). Notch activity acts as a sensor for extracellular calcium during vertebrate left-right determination. Nature 427 (6970), 121–128. 10.1038/nature02190 14712268

[B87] Resende R. R. Andrade L. M. Oliveira A. G. Guimarães E. S. Guatimosim S. Leite M. F. (2013). Nucleoplasmic calcium signaling and cell proliferation: calcium signaling in the nucleus. Cell. Commun. Signal 11 (1), 14. 10.1186/1478-811X-11-14 23433362 PMC3599436

[B88] Rodrigues M. A. Gomes D. A. Leite M. F. Grant W. Zhang L. Lam W. (2007). Nucleoplasmic calcium is required for cell proliferation. J. Biol. Chem. 282 (23), 17061–17068. 10.1074/jbc.M700490200 17420246 PMC2825877

[B89] Rodriguez-Mora O. G. LaHair M. M. McCubrey J. A. Franklin R. A. (2005). Calcium/calmodulin-dependent kinase I and calcium/calmodulin-dependent kinase kinase participate in the control of cell cycle progression in MCF-7 human breast cancer cells. Cancer Res. 65 (12), 5408–5416. 10.1158/0008-5472.CAN-05-0271 15958590

[B90] Santra P. Amack J. D. (2021). Loss of vacuolar-type H+-ATPase induces caspase-independent necrosis-like death of hair cells in zebrafish neuromasts. Dis. Model. Mech. 14 (7), dmm048997. 10.1242/dmm.048997 34296747 PMC8319552

[B91] Sarmah B. Latimer A. J. Appel B. Wente S. R. (2005). Inositol polyphosphates regulate zebrafish left-right asymmetry. Dev. Cell. 9 (1), 133–145. 10.1016/j.devcel.2005.05.002 15992547

[B92] Schneider I. Houston D. W. Rebagliati M. R. Slusarski D. C. (2008). Calcium fluxes in dorsal forerunner cells antagonize beta-catenin and alter left-right patterning. Development 135 (1), 75–84. 10.1242/dev.004713 18045845

[B93] Schweickert A. Weber T. Beyer T. Vick P. Bogusch S. Feistel K. (2007). Cilia-driven leftward flow determines laterality in Xenopus. Curr. Biol. 17 (1), 60–66. 10.1016/j.cub.2006.10.067 17208188

[B94] Shiratori H. Hamada H. (2014). TGFβ signaling in establishing left-right asymmetry. Semin. Cell. Dev. Biol. 32, 80–84. 10.1016/j.semcdb.2014.03.029 24704359

[B95] Shu X. Huang J. Dong Y. Choi J. Langenbacher A. Chen J. N. (2007). Na,K-ATPase alpha2 and Ncx4a regulate zebrafish left-right patterning. Development 134 (10), 1921–1930. 10.1242/dev.02851 17442698

[B96] Simon V. R. Moran M. F. (2001). SERCA activity is required for timely progression through G1/S. Cell. Prolif. 34 (1), 15–30. 10.1046/j.1365-2184.2001.00192.x 11284916 PMC6495481

[B97] Skelding K. A. Rostas J. A. Verrills N. M. (2011). Controlling the cell cycle: the role of calcium/calmodulin-stimulated protein kinases I and II. Cell. Cycle 10 (4), 631–639. 10.4161/cc.10.4.14798 21301225

[B98] Slusarski D. C. Pelegri F. (2007). Calcium signaling in vertebrate embryonic patterning and morphogenesis. Dev. Biol. 307 (1), 1–13. 10.1016/j.ydbio.2007.04.043 17531967 PMC2729314

[B99] Soofi M. Alpert M. A. Barbadora J. Mukerji B. Mukerji V. (2021). Human laterality disorders: pathogenesis, clinical manifestations, diagnosis, and management. Am. J. Med. Sci. 362 (3), 233–242. 10.1016/j.amjms.2021.05.020 34052215

[B100] Takao D. Nemoto T. Abe T. Kiyonari H. Kajiura-Kobayashi H. Shiratori H. (2013). Asymmetric distribution of dynamic calcium signals in the node of mouse embryo during left-right axis formation. Dev. Biol. 376 (1), 23–30. 10.1016/j.ydbio.2013.01.018 23357539

[B101] Tanaka Y. Morozumi A. Hirokawa N. (2023). Nodal flow transfers polycystin to determine mouse left-right asymmetry. Dev. Cell. 58 (16), 1447–1461 e6. 10.1016/j.devcel.2023.06.002 37413993

[B102] Tanaka Y. Okada Y. Hirokawa N. (2005). FGF-induced vesicular release of Sonic hedgehog and retinoic acid in leftward nodal flow is critical for left-right determination. Nature 435 (7039), 172–177. 10.1038/nature03494 15889083

[B104] Tay H. G. Schulze S. K. Compagnon J. Foley F. C. Heisenberg C. P. Yost H. J. (2013). Lethal giant larvae 2 regulates development of the ciliated organ Kupffer's vesicle. Development 140 (7), 1550–1559. 10.1242/dev.087130 23482490 PMC3596994

[B105] Thompson M. Andrade V. A. Andrade S. J. Pusl T. Ortega J. M. Goes A. M. (2003). Inhibition of the TEF/TEAD transcription factor activity by nuclear calcium and distinct kinase pathways. Biochem. Biophys. Res. Commun. 301 (2), 267–274. 10.1016/s0006-291x(02)03024-3 12565854

[B107] Warga R. M. Kane D. A. (2018). Wilson cell origin for kupffer's vesicle in the zebrafish. Dev. Dyn. 247 (9), 1057–1069. 10.1002/dvdy.24657 30016568

[B108] Yoshiba S. Hamada H. (2014). Roles of cilia, fluid flow, and Ca2+ signaling in breaking of left-right symmetry. Trends Genet. 30 (1), 10–17. 10.1016/j.tig.2013.09.001 24091059

[B110] Yuan S. Zhao L. Brueckner M. Sun Z. (2015). Intraciliary calcium oscillations initiate vertebrate left-right asymmetry. Curr. Biol. 25 (5), 556–567. 10.1016/j.cub.2014.12.051 25660539 PMC4469357

[B111] Zhang M. Zhang J. Lin S. C. Meng A. (2012). β-Catenin 1 and β-catenin 2 play similar and distinct roles in left-right asymmetric development of zebrafish embryos. Development 139 (11), 2009–2019. 10.1242/dev.074435 22535411

[B112] Zhang S. J. Zou M. Lu L. Lau D. Ditzel D. A. W. Delucinge-Vivier C. (2009). Nuclear calcium signaling controls expression of a large gene pool: identification of a gene program for acquired neuroprotection induced by synaptic activity. PLoS Genet. 5 (8), e1000604. 10.1371/journal.pgen.1000604 19680447 PMC2718706

