# Supplementary material for: Calcium signaling mediates proliferation of the precursor cells that give rise to the ciliated left-right organizer in the zebrafish embryo
Source: Front Mol Biosci. 2023 Dec 12;10:1292076. doi: 10.3389/fmolb.2023.1292076 (PMC10751931; doi:10.3389/fmolb.2023.1292076)
Supplement: Supplementary file 5 [file Table1.DOCX]

**Table S1:** Pharmacological screen targeting pathways previously implicated in DFC development.

| Targeted signaling  pathway | Work implicating pathway in DFCs | Selected drug  and dose | Drug  mechanism | Previous use of drug in zebrafish | Source |
| --- | --- | --- | --- | --- | --- |
| WNT-β-catenin  (activation) | [35, 40, 97, 98] | 10 μM  1-Azakenpaullone  (Az) | Inhibits GSK3B, which degrades β-catenin | [99] | Sigma-Aldrich  (A3734) |
| WNT-β-catenin  (inhibition) | [35, 40, 97, 98] | 30 μM  XAV939 | Tankyrase inhibitor stimulates β-catenin degradation | [100] | Sigma-Aldrich  (X3004) |
| BMP  (inhibition) | [101] | 25 μM  LDN193189 | Inhibits activity of BMP receptors ALK2 and ALK3 | [102] | United States Biological  (366895) |
| FGF  (inhibition) | [103-106] | 25 μM  SU5402 | Inhibits activity of  FGF receptors | [104] | EMD Millipore  (572630) |
| TGFβ  (inhibition) | [29] | 60 μM  SB505142 | Inhibitor of the TGF-β signaling via ALK5 | [107] | ApexBio  (B2289) |
| Ca^2+^ flux  (inhibition) | [40, 41] | 1 μM  Thapsigargin  (Thaps) | Inhibits sarco-endoplasmic reticulum Ca2+ ATPase pump (SERCA), leading to increased cytosolic calcium | [40, 41] | Sigma-Aldrich  (T9033) |
| Notch  (inhibition) | [108, 109] | 100 μM  DAPT | Inhibits γ-secretase, preventing the release of Notch intracellular domain | [110] | TOCRIS  (2634) |
| mTOR  (inhibition) | [111, 112] | 1 μM  Rapamycin  (Rap) | Inhibits mTORC1 complex | [111] | Cayman Chemical Company  (13346) |
| SHH  (activation) | [113] | 10 μM  Smoothened agonist (SAG) | Activates smoothened receptor | [114] | EMD Millipore  (566661) |
| Ca^2+^ flux  (inhibition) | [40, 41] | 100 μM Cyclopizionic acid  (CPA) | Inhibits sarco-endoplasmic reticulum Ca2+ ATPase pump (SERCA), leading to increased cytosolic calcium | [40, 41] | EMD Millipore  (239805) |
