# Supplementary material for: Calcium signaling mediates proliferation of the precursor cells that give rise to the ciliated left-right organizer in the zebrafish embryo
Source: Front Mol Biosci. 2023 Dec 12;10:1292076. doi: 10.3389/fmolb.2023.1292076 (PMC10751931; doi:10.3389/fmolb.2023.1292076)
Supplement: Supplementary file 6 [file Table8.DOCX]

| Embryo | Starting  stage | Imaging  interval | Imaging duration | Observed cytoplasmic Ca^2+^ flux | Observed nuclear  Ca^2+^ flux | Calculated cytoplasmic  Ca^2+^ fluxes/hour | Calculated  nuclear  Ca^2+^ fluxes/hour |
| --- | --- | --- | --- | --- | --- | --- | --- |
| DMSO #1 | 70% epiboly | 15 sec | 10 min | 3 | 1 | 18 | 6 |
| DMSO #2 | 70% epiboly | 15 sec | 10 min | 3 | 0 | 18 | 0 |
| DMSO #3 | 70% epiboly | 15 sec | 10 min | 3 | 0 | 18 | 0 |
| DMSO #4 | 70% epiboly | 15 sec | 10 min | 3 | 1 | 18 | 6 |
| DMSO #5 | 70% epiboly | 15 sec | 10 min | 0 | 1 | 0 | 6 |
| DMSO #6 | 70% epiboly | 15 sec | 10 min | 2 | 0 | 12 | 0 |
| DMSO #7 | 70% epiboly | 15 sec | 10 min | 1 | 0 | 6 | 0 |
| DMSO #8 | 70% epiboly | 15 sec | 10 min | 4 | 1 | 24 | 6 |
| DMSO #9 | 70% epiboly | 15 sec | 10 min | 3 | 1 | 18 | 6 |
| DMSO #10 | 70% epiboly | 15 sec | 10 min | 2 | 0 | 12 | 0 |
| DMSO #11 | 70% epiboly | 15 sec | 10 min | 9 | 0 | 54 | 0 |
| DMSO #12 | 70% epiboly | 15 sec | 10 min | 4 | 0 | 24 | 0 |
|  |  |  |  |  | **Avg** | **18.5** | **2.5** |
|  |  |  |  |  | *sd* | *13.2* | *3.1* |
|  |  |  |  |  |  |  |  |
| Thaps #1 | 70% epiboly | 15 sec | 10 min | 0 | 0 | 0 | 0 |
| Thaps #2 | 70% epiboly | 15 sec | 10 min | 0 | 0 | 0 | 0 |
| Thaps #3 | 70% epiboly | 15 sec | 10 min | 0 | 0 | 0 | 0 |
| Thaps #4 | 70% epiboly | 15 sec | 10 min | 0 | 0 | 0 | 0 |
| Thaps #5 | 70% epiboly | 15 sec | 10 min | 0 | 0 | 0 | 0 |
| Thaps #6 | 70% epiboly | 15 sec | 10 min | 0 | 0 | 0 | 0 |
|  |  |  |  |  | **Avg** | **0** | **0** |
|  |  |  |  |  |  |  |  |
| CPA #1 | 70% epiboly | 15 sec | 10 min | 0 | 0 | 0 | 0 |
| CPA #2 | 70% epiboly | 15 sec | 10 min | 0 | 0 | 0 | 0 |
| CPA #3 | 70% epiboly | 15 sec | 10 min | 0 | 0 | 0 | 0 |
| CPA #4 | 70% epiboly | 15 sec | 10 min | 0 | 0 | 0 | 0 |
| CPA #5 | 70% epiboly | 15 sec | 10 min | 0 | 0 | 0 | 0 |
| CPA #6 | 70% epiboly | 15 sec | 10 min | 0 | 0 | 0 | 0 |
|  |  |  |  |  | **Avg** | **0** | **0** |

**Table S8.** Analysis of the frequency of Ca^2+^ flux events in DFCs in DMSO, Thapsigargin or cyclopiazonic acid (CPA) treated embryos.

Avg=average

sd=one standard deviation
