# Supplementary material for: Calcium signaling mediates proliferation of the precursor cells that give rise to the ciliated left-right organizer in the zebrafish embryo
Source: Front Mol Biosci. 2023 Dec 12;10:1292076. doi: 10.3389/fmolb.2023.1292076 (PMC10751931; doi:10.3389/fmolb.2023.1292076)
Supplement: Supplementary file 7 [file Table7.DOCX]

|  | Cytoplasmic  Ca^2+^flux events |  |  |  |  | Nuclear  Ca^2+^flux events |  |  |  |
| --- | --- | --- | --- | --- | --- | --- | --- | --- | --- |
| Quadrant: | **LA** | **RA** | **LP** | **RP** |  | **LA** | **RA** | **LP** | **RP** |
| Embryo A | 3 | 0 | 2 | 0 |  | 0 | 1 | 3 | 3 |
| Embryo B | 1 | 1 | 0 | 2 |  | 2 | 0 | 0 | 0 |
| Embryo C | 3 | 1 | 1 | 0 |  | 1 | 1 | 0 | 1 |
| Embryo D | 1 | 0 | 3 | 1 |  | 0 | 0 | 1 | 0 |
| Embryo E | 0 | 0 | 1 | 3 |  | 1 | 0 | 0 | 0 |
| Embryo F | 4 | 0 | 2 | 0 |  | 2 | 0 | 0 | 0 |
| Embryo G | 0 | 1 | 0 | 5 |  | 0 | 0 | 0 | 0 |
| Embryo H | 2 | 3 | 2 | 2 |  | 0 | 0 | 0 | 2 |
| Embryo I | 4 | 3 | 2 | 2 |  | 1 | 2 | 0 | 0 |
| Embryo J | 0 | 0 | 2 | 1 |  | 0 | 0 | 1 | 0 |
| Total | **18** | **9** | **14** | **15** |  | **7** | **4** | **5** | **6** |
| *%* | ***32%*** | ***16%*** | ***25%*** | ***27%*** |  | ***32%*** | ***18%*** | ***23%*** | ***27%*** |

**Table S7.** Spatial mapping of Ca^2+^ flux events in DFCs in ten embryos.

LA=left-anterior

RA=right-anterior

LP=left-posterior

RP=right-posterior
