# Supplementary material for: Calcium signaling mediates proliferation of the precursor cells that give rise to the ciliated left-right organizer in the zebrafish embryo
Source: Front Mol Biosci. 2023 Dec 12;10:1292076. doi: 10.3389/fmolb.2023.1292076 (PMC10751931; doi:10.3389/fmolb.2023.1292076)
Supplement: Supplementary file 8 [file Table9.DOCX]

| Ca^2+^ flux  event | Tracked  DFC | Starting  stage | Imaging  interval | Imaging duration | Observed  outcome |
| --- | --- | --- | --- | --- | --- |
| **whole cytoplasm** | #1 | 60% epiboly | 30 sec | 20 min | No change  after 20 min |
|  | #2 | 60% epiboly | 15 sec | 10 min | No change  after 9.5 min |
|  | #3 | 60% epiboly | 15 sec | 13 min | No change  after 12 min |
|  | #4 | 60% epiboly | 15 sec | 10 min | No change  after 6.5 min |
|  | #5 | 60% epiboly | 15 sec | 10 min | No change  after 9.5 min |
|  | #6 | 60% epiboly | 15 sec | 10 min | No change  after 6 min |
|  | #7 | 60% epiboly | 15 sec | 20 min | No change  after 17.5 min |
|  | #8 | 60% epiboly | 15 sec | 20 min | No change  after 17 min |
|  | #9 | 60% epiboly | 15 sec | 20 min | No change  after 19.75 min |
|  | #10 | 60% epiboly | 15 sec | 20 min | No change  after 19.5 min |
| **Nuclear** | #11 | 60% epiboly | 30 sec | 20 min | Cell divided  12 min later |
|  | #12 | 60% epiboly | 30 sec | 20 min | Cell divided  10.5 min later |
|  | #13 | 60% epiboly | 15 sec | 28 min | Cell divided  21 min later |
|  | #14 | 60% epiboly | 30 sec | 20 min | Cell divided  8.5 min later |
|  | #15 | 60% epiboly | 30 sec | 20 min | Cell divided  8.5 min later |
|  | #16 | 70% epiboly | 15 sec | 10 min | Cell did not divide during 10 min imaging |
|  | #17 | 70% epiboly | 30 sec | 54 min | Cell divided  14 min later |
|  | #18 | 60% epiboly | 15 sec | 20 min | Cell divided  12.25 min later |
|  | #19 | 60% epiboly | 15 sec | 20 min | Cell divided  13.25 min later |
|  | #20 | 60% epiboly | 15 sec | 20 min | Cell divided  9.5 min later |

**Table S9.** DFCs tracked following a Ca^2+^ flux event.
