# Supplementary material for: Calcium signaling mediates proliferation of the precursor cells that give rise to the ciliated left-right organizer in the zebrafish embryo
Source: Front Mol Biosci. 2023 Dec 12;10:1292076. doi: 10.3389/fmolb.2023.1292076 (PMC10751931; doi:10.3389/fmolb.2023.1292076)
Supplement: Supplementary file 9 [file Table2.DOCX]

| Group | N | Treatment | Avg # DFCs per embryo | Avg  mitotic index | Total # embryos  analyzed | Total # DFCs  analyzed | Mitotic index  p value | Significant difference? |
| --- | --- | --- | --- | --- | --- | --- | --- | --- |
| 1 | 2 | 1% DMSO | 37.38+9.75 | 0.063+0.058 | 13 | 486 | 0.2807 | no |
|  |  | 25 μM SU5402 | 37.00+9.37 | 0.043+0.025 | 11 | 407 |  |  |
| 2 | 2 | 1% DMSO | 35.17+9.86 | 0.069+0.063 | 12 | 422 | 0.6265 | no |
|  |  | 100 μM DAPT | 34.82+3.74 | 0.058+0.042 | 11 | 383 |  |  |
| 3 | 2 | 1% DMSO | 35.22+13.65 | 0.041+0.037 | 9 | 317 | 0.5714 | no |
|  |  | 10 μM Az | 26.80+5.49 | 0.031+0.033 | 10 | 250 |  |  |
| 4 | 3 | 1% DMSO | 31.27+11.58 | 0.041+0.049 | 15 | 469 | 0.1910 | no |
|  |  | 30 μM XAV939 | 30.75+11.43 | 0.022+0.024 | 16 | 492 |  |  |
| 5 | 2 | 1% DMSO | 34.20+9.78 | 0.069+0.063 | 12 | 422 | 0.1172 | no |
|  |  | 1 μM Rap | 36.80+10.33 | 0.035+0.030 | 10 | 357 |  |  |
| 6 | 2 | 1% DMSO | 37.38+9.75 | 0.063+0.058 | 13 | 486 | 0.2478 | no |
|  |  | 25 μM LDN93189 | 27.08+9.07 | 0.039+0.040 | 12 | 325 |  |  |
| 7 | 2 | 1% DMSO | 38.33+15.64 | 0.05+0.034 | 12 | 548 | 0.0005 | yes |
|  |  | 1 μM Thaps | 34.17+9.23 | 0.00+0.00 | 16 | 363 |  |  |
| 8 | 2 | 1% DMSO | 33.50+5.68 | 0.031+0.028 | 8 | 268 | 0.8115 | no |
|  |  | 60 μM SB505142 | 42.55+10.43 | 0.035+0.034 | 11 | 468 |  |  |
| 9 | 2 | 1% DMSO | 34.20+9.78 | 0.069+0.063 | 12 | 422 | 0.0925 | no |
|  |  | 10 μM SAG | 37.71+5.25 | 0.033+0.030 | 11 | 413 |  |  |

**Table S2.** Results from a pharmacological screen to identify regulators of DFC mitosis.

N=Number of independent trials

An unpaired two-tailed t-test with Welch’s correction was used for statistical analysis
