# Supplementary material for: Calcium signaling mediates proliferation of the precursor cells that give rise to the ciliated left-right organizer in the zebrafish embryo
Source: Front Mol Biosci. 2023 Dec 12;10:1292076. doi: 10.3389/fmolb.2023.1292076 (PMC10751931; doi:10.3389/fmolb.2023.1292076)
Supplement: Supplementary file 10 [file DataSheet1.pdf]

Supplemental Figure and Movie Legends

Fig. S1. SERCA inhibitor treatments do not alter the mitotic index of non-DFCs at the dorsal margin. (**A**) Representative merged images of pHH3 staining in *Tg(sox17:EGFP-caax)* embryos treated with 1% DMSO (control) or 1 µM Thaps at 60% epiboly for 60 min. (**B**) Bar graphs indicate average mitotic index of neighboring non-DFCs in DMSO (control) and 1 µM Thaps treated embryos and error bars represent one standard deviation. Each circle on the graph represents results from an individual embryo. An unpaired two-tailed t-test with Welch's correction was used for statistical analysis. ns=not significant ($p=0.4446$).

Fig. S2. SERCA inhibitor treatments during epiboly alters heart looping. (**A-B**) Embryos at 2 days post-fertilization that were treated with 1% DMSO (control) (A) or 1 µM thapsigargin (B) at 60% epiboly for 60 min. (**C-E**) Representative images of normal rightward looping of the heart (arrow) in a control embryo (C), and midline (D) or reversed (E) looping in thapsigargin treated embryos. The heart was labeled by EGFP expression in cardiomyocytes. V=ventricle; A=atrium. (**F**) Rightward heart looping was observed in most control embryos, whereas the heart often remained along the midline or looped to the left in thapsigargin treated embryos. n=number of embryos analyzed.

Fig. S3. Spatial location of DFC cytoplasmic Ca^{2+} flux events in individual embryos. Solid colored lines indicate the boundary of the DFC cluster in an individual embryo as determined by confocal images (see **Fig. 6B**). The DFC cluster was divided into quadrants based on the overall length and height of the cluster. Colored ovals indicate the location of cytoplasmic Ca^{2+} flux events. Dashed lines group clusters of DFC cytoplasmic Ca^{2+} flux events. The DFC cluster for all 5 embryos were overlayed to create **Fig. 6C**. L=left, R=right, A=anterior, P=posterior.

Fig. S4. Spatial location of DFC nuclear Ca^{2+} fluxes in individual embryos. (**A**) Solid colored lines indicate the boundary of the DFC cluster in an individual embryo as determined by confocal images (see **Fig. 7B**). The DFC cluster was divided into quadrants based on the overall length and height of the cluster. Colored ovals indicate the location of nuclear Ca^{2+} flux events. The DFC cluster for all 5 embryos were overlayed to create **Fig. 7C**. (**B-D**) Location of DFC nuclear Ca^{2+} flux events in DFC quadrants (B), along the LR axis (C), and along the AP axis (D). L=left, R=right, A=anterior, P=posterior.

Movie S1. Visualization of Ca^{2+} dynamics in a *Tg(act2b:GCaMP6f)* embryonic heart. Confocal optical section through the beating embryonic heart detects transient increases in Ca^{2+} concentration (GCaMP6f fluorescence intensity) in cardiomyocytes visualized using the 16-colors lookup table (FIJI software).

Movie S2. Visualization of Ca^{2+} dynamics in a *Tg(act2b:GCaMP6f)* embryonic olfactory pit. Confocal imaging of the embryonic olfactory pit detects transient

increases in Ca^{2+} concentration (GCaMP6f fluorescence intensity) visualized using the 16-colors lookup table (FIJI software).

Movie S3. Visualization of Ca^{2+} dynamics in a *Tg(act2b:GCaMP6f)* embryonic neuromast. Confocal imaging of an embryonic neuromast detects transient increases in Ca^{2+} concentration (GCaMP6f fluorescence intensity) visualized using the 16-colors lookup table (FIJI software).

Movie S4. Visualization of Ca^{2+} dynamics in DFCs. Time-lapse confocal imaging of Ca^{2+} flux events in wild-type *Tg(act2b:GCaMP6f)*; *Tg(sox17:EGFP-caax)* embryos starting at the 60% epiboly stage. DFC cell membranes are labeled by *Tg(sox17:EGFP-caax)* expression, and changes in Ca^{2+} concentration (GCaMP6f fluorescence intensity) are visualized using the cyan hot lookup table (FIJI software). This is a maximum projection of the entire DFC cluster.Timestamp= hours:minutes:seconds:milliseconds.

Movie S5. Visualization of Ca^{2+} dynamics in DMSO control treated DFCs. Time-lapse confocal imaging of Ca^{2+} flux events in *Tg(act2b:GCaMP6f)*; *Tg(sox17:EGFP-caax)* embryos treated with 1% DMSO at the 60% epiboly stage for 60 min. DFCs were imaged at at the 70% epiboly stage. DFC cell membranes are labeled by *Tg(sox17:EGFP-caax)* expression, and changes in Ca^{2+} concentration (GCaMP6f fluorescence intensity) are visualized using the cyan hot lookup table (FIJI software). This is a maximum projection of the entire DFC cluster. Timestamp= hours:minutes:seconds:milliseconds.

Movie S6. Visualization of Ca^{2+} dynamics in thapsigargin treated DFCs. Time-lapse confocal imaging of Ca^{2+} flux events in *Tg(act2b:GCaMP6f)*; *Tg(sox17:EGFP-caax)* embryos treated with 1 μM thapsigargin at the 60% epiboly stage for 60 min. DFCs were imaged at at the 70% epiboly stage. DFC cell membranes are labeled by *Tg(sox17:EGFP-caax)* expression, and changes in Ca^{2+} concentration (GCaMP6f fluorescence intensity) are visualized using the cyan hot lookup table (FIJI software). This is a maximum projection of the entire DFC cluster. Timestamp= hours:minutes:seconds:milliseconds.

Movie S7. Visualization of Ca^{2+} dynamics in cyclopiazonic acid treated DFCs. Time-lapse confocal imaging of Ca^{2+} flux events in *Tg(act2b:GCaMP6f)*; *Tg(sox17:EGFP-caax)* embryos treated with 100 μM cyclopiazonic acid at the 60% epiboly stage for 60 min. DFCs were imaged at at the 70% epiboly stage. DFC cell membranes are labeled by *Tg(sox17:EGFP-caax)* expression, and changes in Ca^{2+} concentration (GCaMP6f fluorescence intensity) are visualized using the cyan hot lookup table (FIJI software). This is a maximum projection of the entire DFC cluster. Timestamp= hours:minutes:seconds:milliseconds.

Movie S8. DFC behavior following cytoplasmic Ca^{2+} fluxes. The behavior of a DFC (arrow) in a *Tg(act2b:GCaMP6f)*; *Tg(sox17:EGFP-caax)* embryo that undergoes multiple cytoplasmic Ca^{2+} flux events (asterisks) was tracked for 20 min. These cytoplasmic Ca^{2+} flux events did not result in gross changes in DFC morphology, behavior, or position in

this timeframe. This is a single focal plane within the DFC cluster. Timestamp= minutes:seconds.

Movie S9. DFC mitosis following nuclear Ca^{2+} flux. The behavior of a DFC (arrow) in a *Tg(act2b:GCaMP6f)*; *Tg(sox17:EGFP-caax)* embryo that undergoes a nuclear Ca^{2+} flux event (asterisk) was tracked for 20 min. The DFC rounded up and divided into two daughter cells. This is a single focal plane within the DFC cluster. Timestamp= minutes:seconds.
