# Supplementary material for: Calcium signaling mediates proliferation of the precursor cells that give rise to the ciliated left-right organizer in the zebrafish embryo
Source: Front Mol Biosci. 2023 Dec 12;10:1292076. doi: 10.3389/fmolb.2023.1292076 (PMC10751931; doi:10.3389/fmolb.2023.1292076)
Supplement: Supplementary file 11 [file Table5.DOCX]

| DFC | Starting  stage | Imaging  interval | Imaging duration | Start of cytoplasmic Ca^2+^ flux | End of cytoplasmic  Ca^2+^ flux | Duration of cytoplasmic  Ca^2+^ flux |
| --- | --- | --- | --- | --- | --- | --- |
| #1 | 60% epiboly | 1 sec | 1 min | 45 sec | 60 sec | 15 sec |
| #2 | 60% epiboly | 1 sec | 1 min | 10 sec | 25 sec | 15 sec |
| #3 | 60% epiboly | 1 sec | 1 min | 30 sec | 40 sec | 10 sec |
| #4 | 60% epiboly | 1 sec | 1 min | 38 sec | 53 sec | 15 sec |
| #5 | 60% epiboly | 1 sec | 1 min | 25 sec | 38 sec | 13 sec |
| #6 | 60% epiboly | 1 sec | 1 min | 35 sec | 44 sec | 9 sec |
| #7 | 60% epiboly | 1 sec | 1 min | 24 sec | 36 sec | 12 sec |
| #8 | 60% epiboly | 1 sec | 1 min | 44 sec | 56 sec | 12 sec |
| #9 | 60% epiboly | 1 sec | 1 min | 51 sec | 59 sec | 8 sec |
| #10 | 60% epiboly | 1 sec | 1 min | 37 sec | 42 sec | 5 sec |
| #11 | 60% epiboly | 1 sec | 1 min | 52 sec | 61 sec | 9 sec |
| #12 | 60% epiboly | 1 sec | 1 min | 20 sec | 32 sec | 12 sec |
| #13 | 60% epiboly | 1 sec | 1 min | 36 sec | 53 sec | 17 sec |
|  |  |  |  |  | **Avg** | **11.7 sec** |
|  |  |  |  |  | *sd* | *3.4 sec* |

**Table S5.** Analysis of cytoplasmic Ca^2+^ flux duration in DFCs.

Avg=average

sd=one standard deviation
