# Supplementary material for: Calcium signaling mediates proliferation of the precursor cells that give rise to the ciliated left-right organizer in the zebrafish embryo
Source: Front Mol Biosci. 2023 Dec 12;10:1292076. doi: 10.3389/fmolb.2023.1292076 (PMC10751931; doi:10.3389/fmolb.2023.1292076)
Supplement: Supplementary file 12 [file Table3.DOCX]

| Group | N | Treatment | Avg #  DFCs  per embryo | Avg  mitotic  index | Total # embryos  analyzed | Total # DFCs  analyzed | Mitotic index  p value | Significant difference  ? |
| --- | --- | --- | --- | --- | --- | --- | --- | --- |
| 1 | 2 | 1% DMSO | 34.25+10.32 | 0.068+0.044 | 16 | 548 | <0.0001 | yes |
|  |  | 1 μM Thaps | 36.00+11.71 | 0.005+0.012 | 11 | 396 |  |  |
|  |  |  |  |  |  |  |  |  |
| 2 | 3 | 1% DMSO | 33.72+10.21 | 0.054+0.047 | 18 | 607 | 0.0002 | yes |
|  |  | 100 μM CPA | 29.92+5.81 | 0.002+0.008 | 13 | 389 |  |  |

**Table S3.** SERCA inhibitor treatments reduce the mitotic index of DFCs.

N=Number of independent trials

An unpaired two-tailed t-test with Welch’s correction was used for statistical analysis
