# Supplementary material for: Calcium signaling mediates proliferation of the precursor cells that give rise to the ciliated left-right organizer in the zebrafish embryo
Source: Front Mol Biosci. 2023 Dec 12;10:1292076. doi: 10.3389/fmolb.2023.1292076 (PMC10751931; doi:10.3389/fmolb.2023.1292076)
Supplement: Supplementary file 14 [file Table4.DOCX]

| Developmental stage | Treatment | Avg  % of DFCs in G1 | Avg  % of DFCs in S/G2/M | Total # embryos  analyzed | N | G1  t-test  p value | Significant difference  ? |
| --- | --- | --- | --- | --- | --- | --- | --- |
| 75% epiboly | 1% DMSO | 44+18% | 56+18% | 12 | 3 | 0.9388 | no |
|  | 1 μM Thaps | 43+17% | 57+17% | 18 | 3 |  |  |
|  |  |  |  |  |  |  |  |
| 90% epiboly | 1% DMSO | 75+12% | 25+12% | 13 | 3 | 0.0001 | yes |
|  | 1 μM Thaps | 52+15% | 48+15% | 16 | 3 |  |  |

**Table S4.** Impact of thapsigargin treatment on DFC cell cycle.

N=Number of independent trials

An unpaired two-tailed t-test with Welch’s correction was used for statistical analysis
