# Supplementary material for: Calcium signaling mediates proliferation of the precursor cells that give rise to the ciliated left-right organizer in the zebrafish embryo
Source: Front Mol Biosci. 2023 Dec 12;10:1292076. doi: 10.3389/fmolb.2023.1292076 (PMC10751931; doi:10.3389/fmolb.2023.1292076)
Supplement: Supplementary file 15 [file Table6.DOCX]

| Embryo | Starting  stage | Imaging  interval | Imaging duration | # Observed cytoplasmic Ca^2+^ flux | # Observed nuclear  Ca^2+^ flux | Calculated cytoplasmic Ca^2+^ fluxes/hour | Calculated  nuclear  Ca^2+^ fluxes/hour |
| --- | --- | --- | --- | --- | --- | --- | --- |
| #1 | 60% epiboly | 15 sec | 20 min | 6 | 2 | 18 | 6 |
| #2 | 60% epiboly | 15 sec | 10 min | 1 | 1 | 6 | 6 |
| #3 | 60% epiboly | 15 sec | 30 min | 7 | 1 | 14 | 2 |
| #4 | 60% epiboly | 15 sec | 10 min | 1 | 0 | 6 | 0 |
| #5 | 60% epiboly | 15 sec | 10 min | 5 | 1 | 30 | 6 |
| #6 | 60% epiboly | 15 sec | 10 min | 4 | 1 | 24 | 6 |
| #7 | 60% epiboly | 15 sec | 10 min | 1 | 0 | 6 | 0 |
| #8 | 60% epiboly | 15 sec | 10 min | 7 | 3 | 42 | 18 |
| #9 | 60% epiboly | 15 sec | 20 min | 12 | 3 | 36 | 9 |
|  |  |  |  |  | **Avg** | **20.2** | **5.9** |
|  |  |  |  |  | *sd* | *13.6* | *5.5* |
|  |  |  |  |  |  |  |  |
| #10 | 70% epiboly | 15 sec | 10 min | 3 | 0 | 18 | 6 |
| #11 | 70% epiboly | 15 sec | 10 min | 1 | 1 | 6 | 6 |
| #12 | 70% epiboly | 15 sec | 10 min | 2 | 0 | 12 | 0 |
| #13 | 70% epiboly | 15 sec | 10 min | 6 | 1 | 36 | 6 |
| #14 | 70% epiboly | 15 sec | 10 min | 9 | 0 | 54 | 0 |
| #15 | 70% epiboly | 15 sec | 10 min | 3 | 2 | 18 | 12 |
| #16 | 70% epiboly | 15 sec | 10 min | 2 | 0 | 12 | 0 |
| #17 | 70% epiboly | 15 sec | 10 min | 2 | 0 | 12 | 0 |
|  |  |  |  |  | **Avg** | **21.0** | **3.0** |
|  |  |  |  |  | *sd* | *16.0* | *4.5* |

**Table S6.** Analysis of the frequency of Ca^2+^ flux events in DFCs in wild-type embryos.

Avg=average

sd=one standard deviation
