# Supplementary material for: Calcium signaling mediates proliferation of the precursor cells that give rise to the ciliated left-right organizer in the zebrafish embryo
Source: Front Mol Biosci. 2023 Dec 12;10:1292076. doi: 10.3389/fmolb.2023.1292076 (PMC10751931; doi:10.3389/fmolb.2023.1292076)
Supplement: Supplementary file 23 [file Table10.DOCX]

| Ca^2+^ flux  event | Embryo | Tracked  DFC | Starting  stage | Imaging  interval | Imaging duration | Observed  outcome |
| --- | --- | --- | --- | --- | --- | --- |
| **None** | #1 | #1 | 60% epiboly | 15 sec | 20 min | No change |
|  |  | #2 | 60% epiboly | 15 sec | 20 min | No change |
|  |  | #3 | 60% epiboly | 15 sec | 20 min | No change |
|  |  | #4 | 60% epiboly | 15 sec | 20 min | No change |
|  | #2 | #5 | 60% epiboly | 15 sec | 20 min | No change |
|  |  | #6 | 60% epiboly | 15 sec | 20 min | No change |
|  |  | #7 | 60% epiboly | 15 sec | 20 min | No change |
|  |  | #8 | 60% epiboly | 15 sec | 20 min | No change |
|  | #3 | #9 | 60% epiboly | 15 sec | 16 min | No change |
|  |  | #10 | 60% epiboly | 15 sec | 16 min | No change |
|  |  | #11 | 60% epiboly | 15 sec | 16 min | No change |
|  |  | #12 | 60% epiboly | 15 sec | 16 min | No change |
|  | #4 | #13 | 60% epiboly | 30 sec | 30 min | No change |
|  |  | #14 | 60% epiboly | 30 sec | 30 min | No change |
|  |  | #15 | 60% epiboly | 30 sec | 30 min | No change |
|  |  | #16 | 60% epiboly | 30 sec | 30 min | No change |
|  | #5 | #17 | 60% epiboly | 30 sec | 40 min | No change |
|  |  | #18 | 60% epiboly | 30 sec | 40 min | No change |
|  |  | #19 | 60% epiboly | 30 sec | 40 min | No change |
|  |  | #20 | 60% epiboly | 30 sec | 40 min | No change |

**Table S10.** DFCs tracked that did not experience a Ca^2+^ flux event.
